# Icariin: from preclinical promise to clinical reality—a critical evidence-based assessment

**DOI:** 10.3389/fphar.2026.1832264

**Published:** 2026-09-16

**Authors:** Chengjuan Gu, Yuan Lin, Zhaobo Wang, Mengtian Li, Lin Du, Xingrong Lai, Liping Qiao, Han Wang, Jia Wang, Xiaolin Tong

**Affiliations:** 1 Department of Basic Medical College, Gansu University of Chinese Medicine, Lanzhou, Gansu, China; 2 Shenzhen Hospital, Guangzhou University of Chinese Medicine, Shenzhen, Guangdong, China; 3 The Second Clinical College of Guangzhou University of Chinese Medicine, Guangzhou, China; 4 Guang’anmen Hospital, China Academy of Chinese Medical Sciences, Beijing, China; 5 Beijing University of Chinese Medicine, Beijing, China

**Keywords:** clinical trials, drug delivery systems, Epimedii Folium, icariin, pharmacokinetics, pharmacological effects, safety profiles, structure-activity relationship

## Abstract

Icariin (ICA), the principal flavonoid from Epimedii Folium, aligns with traditional Chinese medicine indications of tonifying kidney-yang and strengthening bone. Prior reviews mostly catalog ICA’s preclinical neuroprotective, anti-fibrotic, anti-tumor, and osteoprotective effects, but lack evidence stratification. Here, we establish an evidence-stratified framework to evaluate ICA’s structure–activity relationships, pharmacokinetics, and safety profiles, explicitly distinguishing preclinical pharmacological actions from unproven clinical efficacy. We delineate translational barriers including interspecies pharmacokinetic gaps, poor bioavailability, and flawed trial designs, and assess delivery systems to address these limitations. This work bridges traditional Chinese medicine practice with graded modern evidence, providing a translational roadmap to guide ICA’s rational clinical development.

## 1 Introduction

Epimedii Folium (Berberidaceae), known commonly as “Yinyanghuo” in traditional Chinese medicine (TCM), is a time-honored botanical drug with a millennia-long history of clinical application. It originates from the dried aerial parts of four officially recognized *Epimedium* species, including *Epimedium brevicornu* Maxim., *Epimedium sagittatum* (Siebold & Zucc.) Maxim., *Epimedium pubescens* Maxim., and *Epimedium koreanum* Nakai. These species are primarily cultivated in Gansu, Jilin, and Sichuan provinces of China. Characterized by pungent and sweet flavors and a warm medicinal property, this botanical drug is traditionally indicated to tonify kidney-Yang, strengthen tendons and bones, and dispel wind-dampness. The traditional formulas of *Epimedium* used to treat infertility, fatigue and bone fragility are strongly supported by modern pharmacological evidence of icariin (ICA), which encompasses reproductive regulation and osteoprotective effects. ICA also exerts neuroprotective and anti-fibrotic effects via modulation of inflammatory signaling axes (Lu et al., 2025), explaining the material basis of Epimedii Folium for ameliorating cognitive decline and rheumatoid arthritis in TCM practice. As the core bioactive marker compound of *Epimedium* species, ICA bridges traditional clinical experience and modern pharmacological research, making it the research focus of this ethnopharmacological review (see Figure 1).

**FIGURE 1:**
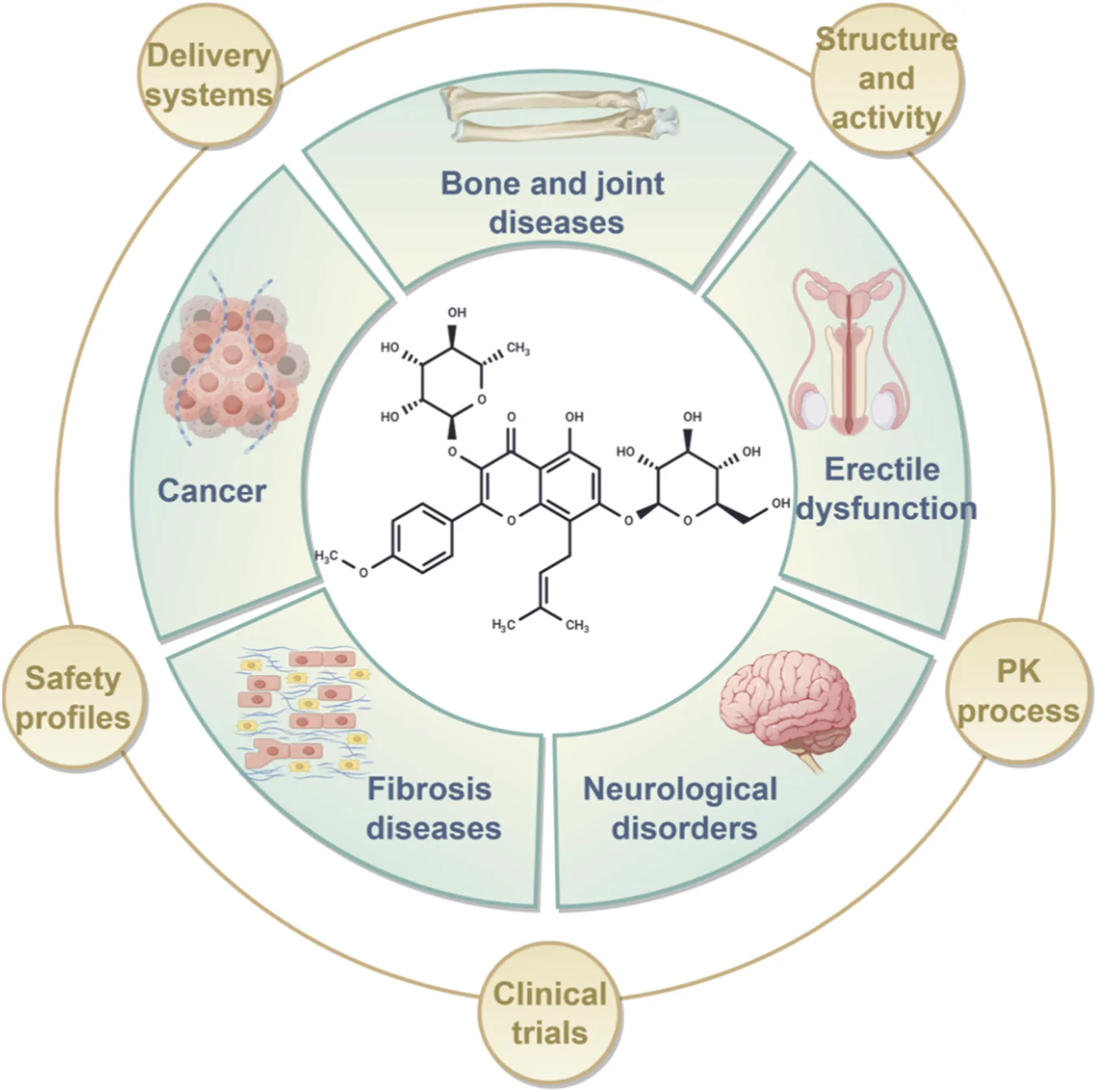
Graphical abstract.

Despite its extensive ethnomedicinal use, the clinical translation of ICA is hampered by unfavorable physicochemical and pharmacokinetic properties. Clarifying ICA’s structure–activity relationship facilitates the understanding of its pharmacokinetic and pharmacodynamic characteristics. ICA features a polar diglycoside moiety at the C-3 and C-7 positions, which severely restricts passive transmembrane diffusion across the intestinal epithelium and blood-brain barrier. Consequently, the intact prototype exhibits negligible systemic bioavailability. Its *in vivo* bioactivity relies predominantly on gut microbiota-mediated deglycosylation to active aglycones, such as icariside II (ICA II) and icaritin (ICT) (Han et al., 2022). Notably, this biotransformation is highly dependent on the route of administration (Cheng et al., 2015) and exhibits significant interspecies differences. Structurally, the prenyl group at the C-8 position imparts enhanced osteogenic potency (Ming et al., 2013). While traditional sheep suet processing is empirically employed in TCM to attenuate toxicity and reinforce efficacy, modern mechanistic validation of these effects remains insufficient. Furthermore, comprehensive safety assessments, particularly regarding developmental toxicity and long-term oral exposure, are warranted. Critically, ICA and its metabolites modulate cytochrome P450 (CYP) enzymes (Chen Y. et al., 2019) and P-glycoprotein (P-gp) (Zhang et al., 2012), posing a potential risk for botanical-drug interactions through alterations in the systemic clearance of co-administered medications.

While previous reviews have separately summarized research advances regarding Epimedii Folium and ICA focusing on botany (Wang Y. et al., 2025), osteoporosis (Fu et al., 2025), organ fibrosis (Zhao and Zhang, 2025), reproductive disorders (Liu et al., 2025), neurodegeneration (Rodrigues-Soares et al., 2026), and drug delivery technologies. Departing from descriptive summaries, this review establishes an evidence-stratified framework, critically bridging ICA’s preclinical promise and clinical reality. Grounded in this perspective, the present work integrates ethnopharmacology with modern pharmacology from three innovative angles: First, we transcend simplistic cataloging by linking the canonical TCM indications of *Epimedii Folium* to the multi-target pharmacological effects of ICA, conducting a comprehensive, stratified evidence-based assessment. Second, we delineate of ICA’s pharmacokinetic profiles, characterized by low oral bioavailability, prominent interspecies discrepancies, and gut microbiota-dependent metabolism, while evaluating advanced delivery strategies designed to bridge the PK-translation gap. Third, and most critically, we explicitly delineate the mismatches between encouraging preclinical findings and clinical outcomes, reviewing safety data, potential botanical-drug interaction risks, and core challenges restricting bench-to-bedside translation.

A comprehensive literature search was performed across PubMed, Scopus, Web of Science, and Google Scholar, covering publications dated from October 1998 to 31 May 2026 (detailed search strategies are provided in Supplementary File S1). Records were screened to exclude retracted or non-peer-reviewed articles. Following rigorous screening of titles, abstracts, and full-text content, 168 eligible publications were ultimately enrolled in this review. Notably, pharmacological and safety evaluations were restricted to studies reporting compound purity and verified experimental sources. To objectively assess data reliability, we implemented a three-tiered evidence classification:

Level A (Strong evidence): supported by *in vitro*, animal, and clinical data.
Level B (Moderate evidence): supported by *in vitro* and animal data. No clinical data yet.
Level C (Preliminary evidence): supported by *in vitro* studies. Or *in silico* prediction without experimental validation.

The central panel depicts the chemical skeleton of ICA. The inner ring summarizes its major therapeutic indications, including bone and joint diseases, erectile dysfunction, neurological disorders, fibrotic diseases, and cancer. The outer ring delineates core research domains encompassing structure–activity relationships, pharmacokinetics, safety profiles, clinical trials, and delivery systems.

## 2 Chemistry and pharmacokinetics of icariin

### 2.1 Structure and activity relationships (preliminary evidence)

ICA (C_33_H_40_O_15_, molecular weight: 676.67 g/mol) is a characteristic 8-prenylated kaempferol 3-O-rhamnoside-7-O-glucoside isolated from multiple *Epimedium* species. As a crystalline solid, it remains stable for up to 2 years when stored at −20 °C. ICA exhibits favorable solubility in organic solvents such as ethanol but presents poor aqueous solubility (Wang et al., 2012). Its core scaffold consists of a kaempferol backbone substituted with a C-8-γ,γ-dimethylallyl (prenyl) group, a free 5-hydroxyl group, a 3-O-α-L-rhamnopyranosyl moiety and a 7-O-β-D-glucopyranosyl moiety (see Figure 2). The C-8 prenyl substituent is essential for anti-tumor, anti-inflammatory, and phosphodiesterase 5 (PDE5) inhibitory activities (Shi et al., 2021). Moreover, C8-prenylation enhances estrogenic effects by facilitating ligand insertion into the hydrophobic binding pocket of estrogen receptor α (ERα), thereby underpinning ICA’s efficacy in managing reproductive disorders (Mbachu et al., 2020). By contrast, the 3-O-rhamnosyl and 7-O-glucosyl glycosidic moieties elevate molecular polarity, restricting passive diffusion across the intestinal epithelium and blood–brain barrier. Molecular docking simulations reveal that the 7-O-glucosyl group forms hydrogen bonds with amino acid residues at the PDE5 active site, while the 3-O-alkanol substituent occupies the enzyme’s hydrophobic catalytic cavity (Chau et al., 2019). In addition, the C2 = C3 double bond and the C4-carbonyl group contribute to ICA’s broad pharmacological properties including antiviral, antibacterial, anticancer, anti-inflammatory, antioxidant, hypoglycemic, neuroprotective, and cardioprotective activities (Wang T. Y. et al., 2018).

**FIGURE 2:**
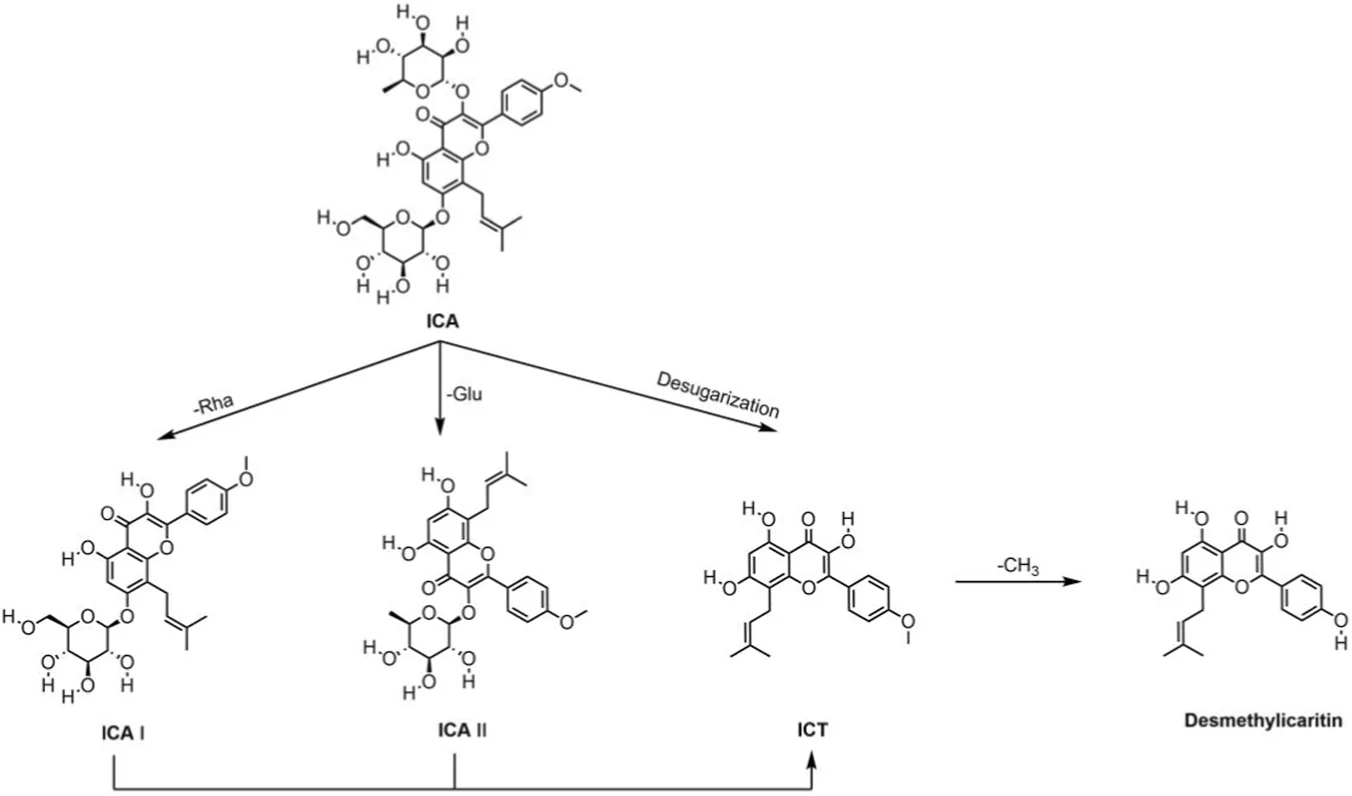
Structural transformation of ICA and its major metabolites.

### 2.2 Pharmacokinetic properties (strong evidence)

To explore interspecies variability, we compared the pharmacokinetic (PK) profiles of ICA in rats (Cheng et al., 2015) (oral and intravenous administration) and mice (Liu W. et al., 2023) (intravenous administration only) (see Table 1). Notable differences in terminal elimination half-life (*t*_1/2_) were observed. The *t*_1/2_ of ICA in rats was around 2.4-fold longer than that in mice. Oral ICA is extensively transformed by gut commensal flora, resulting in large individual variation of *in vivo* drug exposure. Therefore, the above interspecies variability can be partially explained by differences in hepatic clearance capacity as well as intestinal microbiota composition. The poor oral bioavailability of ICA arises from multiple factors, including low aqueous solubility, P-gp-mediated efflux, extensive first-pass metabolism catalyzed by hepatic CYP enzymes and intestinal microbial deglycosylation (Chen et al., 2008b; Zhou et al., 2015; Wu et al., 2010). *In vitro* Caco-2 cell models confirmed P-gp’s pivotal role in ICA efflux (Chen et al., 2008a). Deglycosylation represents the dominant metabolic pathway *in vivo*. Sequential hydrolysis of the 7-O-glucosidic bond yields ICA II, followed by cleavage of the 3-*O*-rhamnosyl moiety to produce ICT (Cheng et al., 2016) (see Figure 2). The area under the plasma concentration–time curve (AUC) value of ICA II is approximately 10-fold higher than parent ICA, confirming that metabolites are the dominant *in vivo* effective substances of oral ICA (Cheng et al., 2015). Consequently, the AUC, rather than *t*_1/2_, serves as a more reliable metric for systemic exposure. Notably, this biotransformation is route-dependent: Oral administration leads to 91.2% conversion of ICA into ICA II, whereas intravenous injection only induces 0.4% metabolite formation (Cheng et al., 2015), highlighting the decisive role of intestinal microbes. Approximately 60% oral ICA is hydrolyzed by intestinal strains secreting β-glucosidase and α-L-rhamnosidase (Park et al., 2008; Li Q. et al., 2022), with dominant contributing genera including *Bacteroides* (Temple et al., 2017), *Lactobacillus* (Shin et al., 2021), and *Bifidobacterium* species (Miao et al., 2025). Following absorption, ICT undergoes hepatic bioactivation via CYP3A-mediated oxidation to form reactive quinone methide derivatives (Chen Y. et al., 2019). However, the specific bacterial strains and precise CYP isoforms involved remain unclarified. ICA distributes widely in metabolically active organs, exhibiting gender-specific accumulation: higher concentrations in the male liver and lung versus female uterus (Xu S. et al., 2017). Biliary excretion mediated by P-gp is the primary elimination pathway of ICA (Wu et al., 2010), with plasma drug concentrations declining rapidly within 4 h post-administration, indicating fast systemic clearance (Wang et al., 2023). While early reports suggested limited blood–brain barrier permeability (Xu S. et al., 2017), recent evidence indicates that appropriately dosed ICA achieves effective hippocampal accumulation, mediating central neuroprotection (Wang et al., 2023).

**TABLE 1:** Key pharmacokinetic parameters of ICA (mean ± SD).

Parameters	Rats		Mouse
	Oral administration (30 mg/kg)	Intravenous injection (30 mg/kg)	Intravenous injection (15 mg/kg)
*t*_1/2_ (min)	73.9 ± 107.4	319.8 ± 339.0	135 ± 120
T_max_ (min)	15.0 ± 0.0	5.0 ± 0.0	1.8 ± 0.0
C_max_ (ng/mL)	27.2 ± 5.4	3.543 × 10^4^ ± 1.026 × 10^4^	1.327 × 10^4^ ± 0.559 × 10^4^
CL (L/min/kg)	46.2 ± 6.0	—	0.21 ± 0.07
MRT_0−t_ (min)	67.9 ± 54.0	27.4 ± 5.9	—
${\text{MRT}}_{0-\infty }$ (min)	83.1 ± 60.8	33.0 ± 14.3	—
AUC_0−t_ (ng/mL × min)	642.7 ± 83.2	6.942 × 10^5^ ± 1.700 × 10^5^	21.658 ± 8.015
${\text{AUC}}_{0-\infty }$ (ng/mL × min)	657.8 ± 80.4	6.954 × 10^5^ ± 1.687 × 10^5^	21.700 ± 8.083

Abbreviations: *t*_1/2_, elimination half-life; T_max_, time to reach maximum concentration; C_max_, maximum plasma concentration; CL, clearance; MRT_0−t_, mean residence time (from 0 to time t); ${\text{MRT}}_{0-\infty }$ , mean residence time (from 0 to infinity); AUC_0−t_, area under the plasma concentration-time curve (from 0 to time t); area under the plasma concentration-time curve (from 0 to infinity).

Human PK data on ICA remain limited. In one human trial with oral ICA (100–1,680 mg daily) from standardized Epimedii Folium capsules, plasma ICA concentrations were below 1 ng/mL in most healthy volunteers. Only one subject in the 1,680 mg group exhibited measurable peaks (2.1 ng/mL at 6 h and 1.6 ng/mL at 8 h) (Brown et al., 2019), directly reflecting negligible oral bioavailability. Another clinical trial reported peak plasma concentrations of 7.89 ± 1.90 ng/mL for ICA and 10.1 ± 5.93 ng/mL for ICA II, with average *t*_1/2_ values of 3 h for both (Zhang et al., 2008). These findings highlight a stark translational disconnect between preclinical PK projections and human exposure.

The schematic depicts ICA metabolism. Sequential deglycosylation converts ICA into icariside I (loss of −Rha) or icariside II (loss of −Glu), with full hydrolysis yielding icaritin (ICT). ICT is further metabolized via O-demethylation (−Me) to desmethylicaritin.

## 3 Pharmacological effects of ICA

The diverse therapeutic effects of Epimedii Folium align coherently with the TCM principles that “kidney governs bone,” and “kidney essence governs reproduction.” Its principal bioactive constituents, ICA and its metabolites, exhibit a broad spectrum of biological activities, including osteoprotection, neuroprotection, amelioration of erectile dysfunction, anti-fibrosis, and anti-tumor effects (see Table 2).

**TABLE 2:** Evidence landscape of the pharmacological effects of ICA and its metabolites.

Pharmacological activities	Indications	Evidence level	Major findings			Current limitations
			Cell models	Animal models	Clinical trials	
Osteoprotective effects	Osteoporosis	B	Osteogenesis ↑; Adipogenesis, osteoclastogenesis and inflammation ↓.	Bone density ↑; Bone turnover ↓	None	Critical gaps persist in both mechanistic depth and clinical translation: preclinical studies lack causal validation through genetic or pharmacological rescue experiments, and clinical corroboration remains notably absent.
	ONFH	B	Osteogenesis ↑; Apoptosis ↓	Preventing bone loss and increasing blood supply	None	
	AIA	B	None	Cartilage degeneration ↓.	None	
	Cancer-related bone destruction	B	Osteoclastogenesis ↓	TRAP-positive areas ↓	None	
Improving erectile dysfunction	Erectile Dysfunction	B	cGMP concentration ↑; PDE5 expression ↓	ICP/MAP ratio, testosterone production and spermatogenesis ↑	None	These findings are constrained by a predominant reliance on rat models, frequent absence of clinical validation, and incomplete mechanistic dissection—particularly regarding direct target binding, functional enzyme activity assays.
Neuroprotective effects	AD	B	Cell viability ↑; LDH release and cell death ↓	Improving cognitive function and autonomous behavior; Aβ deposition ↓	None	These findings are uniformly limited by the absence of clinical validation (except one small pilot trial), frequent lack of incomplete mechanistic dissection, particularly regarding direct target engagement, upstream regulators, and advanced single-cell/synaptic analyses.
	PD	B	None	Motor function, coordination, striatum DA content ↑; Neuronal death ↓	None	
	Stroke	B	None	Neurological scores ↑; Infarct size and brain edema ↓	None	
	Ischemia-related brain injury	B	Neuronal viability ↑; H_2_O_2_-induced neurotoxicity ↓	ICA alone did not obviously improve cognitive impairment	None	
	Spinal cord injury	B	None	Locomotor functional recovery ↑	None	
	Depression	B	None	Depression-like behavior ↓	None	
	BD	A	None	None	Relieving depression and reducing alcohol consumption	
Anti-fibrosis	Pulmonary fibrosis	B	TGF-β1-mediated fibrogenesis ↓	Improving lung histoarchitecture; The collagen deposition and inflammation in lung ↓	None	Findings on fibrosis consistently lack clinical validation in human patients and suffer from mechanistic incompleteness, evidenced by absence of pathway-specific rescue experiments, short follow-up periods, and the limited translational fidelity of chemically induced animal models.
	Liver fibrosis	B	EMT and fibrosis property of HSCs ↓	Liver dysfunction, hepatic steatosis and fibrosis ↓	None	
	Kidney fibrosis	B	Cell autophagy ↑; Activation of renal fibroblasts, pro-inflammatory and fibrotic phenotype of cell ↓	Renal function ↑; Kidney injury and fibrosis ↓	None	
	Cardiac fibrosis	B	Fibrotic phenotype of cell ↓	Improving left ventricular remodeling and myocardial fibrosis	None	
Anti-cancer	Lung cancer	B	Cell apoptosis ↑; Cell proliferation and invasion ↓	Tumor volume and tumor weigh ↓	None	Preclinical studies over-rely on ectopic xenografts, lacking orthotopic relevance, causal rescue experiments, and robust immune profiling. Clinically, evidence is confined to small-scale trials (*n* < 50) and lacks rigorously designed RCTs.
	HCC	A	Cell apoptosis ↑; Cell proliferation, invasion, the immune-suppression in myeloid cells ↓	Tumor growth ↓	46.7% of patients achieved clinical benefit; Compared to ICT monotherapy, combination therapy improved prognosis.	
	Ovarian cancer	B	Cell apoptosis ↑; Cell proliferation, viability, invasiveness and autophagy ↓	Tumor growth ↓	None	
	Prostate cancer	B	Osteoclast differentiation ↓	Tumor growth and bone metastases ↓	None	
	Breast cancer	B	Cell viability and colony formation ↓	Tumor growth and pulmonary metastases ↓	None	
	Colorectal cancer	B	Cell proliferation ↓	Tumor growth ↓	None	
	Breast cancer	B	Cell proliferation, migration, and invasion↓	TNBC growth ↓	None	
	Cervical cancer	B	Cell apoptosis ↑; Cell migration and invasion ↓.	Tumor growth ↓	None	

Abbreviations: MAMs, mitochondria-associated membranes; DA, dopamine; LDH, lactate dehydrogenase; cGMP, cyclic guanosine monophosphate; PDE, phosphodiesterase; ICP/MAP, intracavernous pressure/mean arterial pressure; EMT, epithelial-mesenchymal transition; HCC, hepatocellular carcinoma; RCTs, randomized controlled trials. “↑” indicated the process was upregulated; “↓” indicated the process was downregulated.

### 3.1 Osteoprotective effects

Epimedii Folium is traditionally used to strengthen tendons and bones in TCM, an indication mainly for age-related bone fragility and general physical weakness. Modern pharmacology substantiates that ICA is the primary contributor to this effect. As a non-estrogen alternative agent, ICA ameliorates pathological bone loss by balancing bone formation and resorption cascades (Zhou et al., 2021). It demonstrates efficacy across multiple pathological models, including ovariectomy-induced osteoporosis, dexamethasone (DXM)- and diabetes-associated osteoporosis, glucocorticoid-induced osteonecrosis of the femoral head (ONFH), and arthritis (see Table 3).

**TABLE 3:** Pharmacological effects of ICA in bone diseases.

Indications	Subjects	Control groups	Dose/conc	Pharmacological effects	Key signaling pathways	Current limitations
Osteoporosis (Zou et al., 2024)	*In vivo*: Rats receiving OVX	(NC) Sham	120 mg/kg/d	Counteracting bone turnover (CTX-I***, PINP**, ALP*)	AMPK/mTOR/ULK1; AKT/mTOR/ULK1	Absence of an autophagy inhibitor control group to verify autophagy involvement
Osteoporosis (Ma et al., 2018)	*In vivo*: DXM treated mice	(NC) Saline	10 and 100 mg/kg/d	Inhibiting bone turnover and improving bone microarchitecture (ALP**, TRACP*, OCN* and BMD*)	PI3K/Akt	​Reliance solely on a DXM-induced mouse model limits the generalizability of the findings
Osteoporosis (Qi et al., 2019)	*In vivo*: T1DM rats	(NC) Citrate	100 mg/kg/d	Increasing the bone density and decreasing bone turnover (BMD**, ALP**, CTX-1**, TRACP 5b** and PIPN**)	—	Reliance solely on a STZ-induced T1DM model limits the generalizability of the findings to T2DM osteoporosis
ONFH (Huang et al., 2018)	*In vivo*: Rats treated with lipopolysaccharide and methylprednisolone; *Ex vivo*: BMSCs	*In vivo*: (NC) Saline; *Ex vivo*: (NC) Vehicle	*In vivo*: 30 mg/kg/d; *Ex vivo*: 10^−6^ M	Preventing bone loss and increasing blood supply (BMD*, empty lacunae**, and vessel volume*); Promoting osteogenesis and inhibiting adipogenesis (Runx*, OC*, ALP*, BMP2* and PPARγ*)	PI3K/Akt	Drug regimen lacks pharmacokinetic data to confirm whether effective concentrations reach the femoral head
AIA (Wei et al., 2016)	*In vivo*: Ovalbumin treated rabbits	(NC) No treatment	60 mg/kg/d	Inhibiting cartilage degeneration and improving bone structure (Mankin score* and BMD*)	RANKL	Single-animal-model design limits the generalizability of the findings
ONFH (Yu et al., 2019)	*In vivo*: GIOP induced ONFH rats; *Ex vivo*: BMECs	*In vivo*: (NC) Saline *Ex vivo*: (NC) Vehicle	*In vivo*: 200 mg/kg/d; *Ex vivo*: 10^–5^ M	Improving osteonecrosis (empty lacunae** and vessel volume*); increasing osteogenesis (BMECs viability**, migration** and tube length**)	Akt/Bcl-2	Not evaluating the dose-timing effects of ICA treatment
Osteoporosis (Ho et al., 2018)	*Ex vivo*: UMR-106 cells and MC3T3-E1 cells	(PC) 17β-estradiol; (NC) Vehicle	10^−9^–10^−6^ M	Promoting osteogenesis (cell proliferation rate*, and ALP activities*)	ER signaling; PI3K/Akt	Restricted to immortalized cell lines without validation in primary osteoblasts
Osteoporosis (Li et al., 2018)	*Ex vivo*: rBMSCs	(PC) β-estradiol	10^–6^ M	Increasing osteogenesis and decreasing adipogenesis (rBMSCs activity*, ALP activity*, and the number and size of fat droplets*)	ER/PPAR-γ and C/EBP α	*In vitro* findings in rBMSCs without *in vivo* corroboration
Osteoporosis (Xiao et al., 2014)	*Ex vivo*: UMR-106 cells	(PC) β-estradiol	10^–8^ M	Enhancing cell proliferation and osteogenesis (cell proliferation rate** and ALP activities**)	ER/OPG	Without *in vivo* verification or exploration of the mechanisms mediating ER signaling
ONFH (Chen Y. et al., 2025)	*In vivo*: osteoporosis rats *Ex vivo*: OVX- BMSCs	*In vivo*: (PC) 5- azacytidine *Ex vivo*: (PC) TWS119, and PPT	*In vivo*: 125 mg/kg/d for 12 weeks; *Ex vivo*: 0.1, 1 and 10 μM	Attenuating apoptosis (cell viability rate*** and cell apoptotic rate***)	PI3K/AKT	No validation was performed in human BMSCs, and ERβ participation has not been investigated
Osteoporosis (Zhai et al., 2014)	*Ex vivo*: rBMSCs	(NC) Vehicle	10^–5^ M	Improving osteogenesis (ALP activities**)	PI3K/AKT/Enos/NO/cGMP/PKG	Without *in vivo* validation
Osteoporosis (Hsieh et al., 2011)	*Ex vivo*: Bone marrow cells co-cultured with osteoblasts	(NC) Vehicle	10^−8^ M	Reducing osteoclastogenesis (ALP and TRACP activity**); Inhibiting inflammation (IL-6 and TNF-α**)	MAPKs/NF-κB	Without *in vivo* validation
Cancer-related bone destruction (Kim et al., 2018)	*Ex vivo*: RAW264.7 cells	(NC) Vehicle	10 μM	Impairing osteoclastogenesis and bone resorption (TRACP activity*)	RANKL/TRAF6/ERK1/2 or NF-κB	Reliance on the Raw264.7 cell line without *in vivo* validation
Cancer-related bone destruction (Chen X. et al., 2023)	*In vivo*: Mouse RM1-Luc PCa bone metastasis model; *Ex vivo*: Raw264.7 cells or BMMs	*In vivo*: (NC) Saline *Ex vivo*: (NC) Vehicle	*In vivo*: 20 mg/kg/d; *Ex vivo*: 10–30 µM	Inhibiting cancer bone destruction (TRACP-positive areas*); Inhibiting osteoclast differentiation (TRACP number**)	—	Whether other bone TME components mediate ICA’s pharmacological effects remains to be elucidated
Osteoporosis (Wang Z. et al., 2018)	*Ex vivo*: Rat mandible osteoblasts	(NC) Vehicle	0.15–15 µM	Promoting cell proliferation and differentiation (cell number and ALP activity**)	Wnt/β-catenin	Restricted to transcriptional changes without corroborating protein expression data
Osteoarthritis (Chen et al., 2022)	*Ex vivo*: IL-1β treated SW1353 chondrocytes	(NC) Vehicle	40 μM	Attenuating osteoarthritis (cell viability*, the expression of MMP-3*, and collagen II*)	PI3K/Akt/mTOR	This is an excellent inflammatory model but not a good OA model; lack of positive controls
Osteoporosis (Mok et al., 2010)	*In vivo*: OVX mice; *Ex vivo*: UMR 106 cells	*In vivo*: (PC) β-estradiol *Ex vivo*: (NC) Vehicle	*In vivo*: 0.3 mg/g/d; *Ex vivo*: 10^−10^M	Promoting osteogenesis (cell proliferation * and ALP activity***) and preventing bone loss (total BMD**)	ER/OPG/RANKL	Incomplete mechanistic characterization regarding the upstream kinases mediating ERα phosphorylation and potential ER-independent pathways
Osteoporosis (Liu W. et al., 2024)	*In vivo*: Ketogenic diet mice	(NC) Sham	50 mg/kg/d	Stimulating osteogenesis and reducing bone loss (ALP*, TRAP*, BMD*, OCN*, BV/TV*, Tb.Th*, Tb.N* and Tb.Sp*)	mTOR	Lack of cellular osteoclast validation and incomplete mechanistic dissection of the mTOR pathway
Osteoporosis (Lin et al., 2024)	*In vivo*: Rat ADSC subcutaneous transplantation model; *Ex vivo*: ADSCs	(PC) Resveratrol	1–100 μM	Promoting osteogenesis and inhibiting adipogenesis (PPAR γ** and BrdU*)	Hippo pathway	Restricted to a rat ADSC subcutaneous transplantation model without *in vivo* osteoporosis model validation
Osteoporosis	Late postmenopausal women	(NC) element calcium	60 mg ICA, 15 mg Daidzein, and 3 mg Genistein	Inhibiting bone loss with no detectable endometrial hyperplasia	—	Using a composite extract rather than pure ICA; employing BMD as a surrogate endpoint, and lacking fracture data

Abbreviations: NC, negative control; PC, positive control; BMSC, bone marrow mesenchymal stem cell; BMECs, bone marrow endothelial cells; PPT, propyl pyrazole triol; ADSCs, adipose-derived stem cells. *p < 0.05, **p < 0.01, and ***p < 0.001.

ICA inhibited high bone turnover rate by downregulating the expression of tartrate-resistant acid phosphatase isoform 5b (TRACP-5b) and c-telopeptide of type I collagen (CTX-I) in ovariectomized (OVX) rats (Zou et al., 2024). It enhanced bone microarchitecture, increasing bone volume fraction (BV/TV), trabecular thickness (Tb.Th), and trabecular number (Tb.N), while reducing trabecular separation (Tb.Sp) in DXM mice (Ma et al., 2018). ICA also inhibited bone turnover and increased bone mineral density (BMD) in diabetic models (Qi et al., 2019). In methylprednisolone-induced ONFH, ICA inhibited adipogenic invasion and subchondral bone necrosis, while restoring calcium homeostasis (Huang et al., 2018). ICA attenuated chondrocyte damage and improved trabecular microarchitecture in antigen-induced arthritis (AIA) rabbits (Wei et al., 2016). These effects stem from ICA’s dual modulation of osteoblast and osteoclast activities (Wang Y. et al., 2018). ICA enhanced vascular volume and promoted angiogenesis of bone microvascular endothelial cells (BMECs) (Yu et al., 2019). Unlike estrogen replacement therapy, ICA enhanced BMD and microarchitecture without inducing hypertrophic effects on uterine or mammary tissues, suggesting a superior safety profile (Xue et al., 2012). The molecular underpinnings of these effects, including mTOR1 autophagy signaling, ER signaling, and Wnt/β-catenin activation, are comprehensively discussed in Section 3.6 (see Figure 3).

**FIGURE 3:**
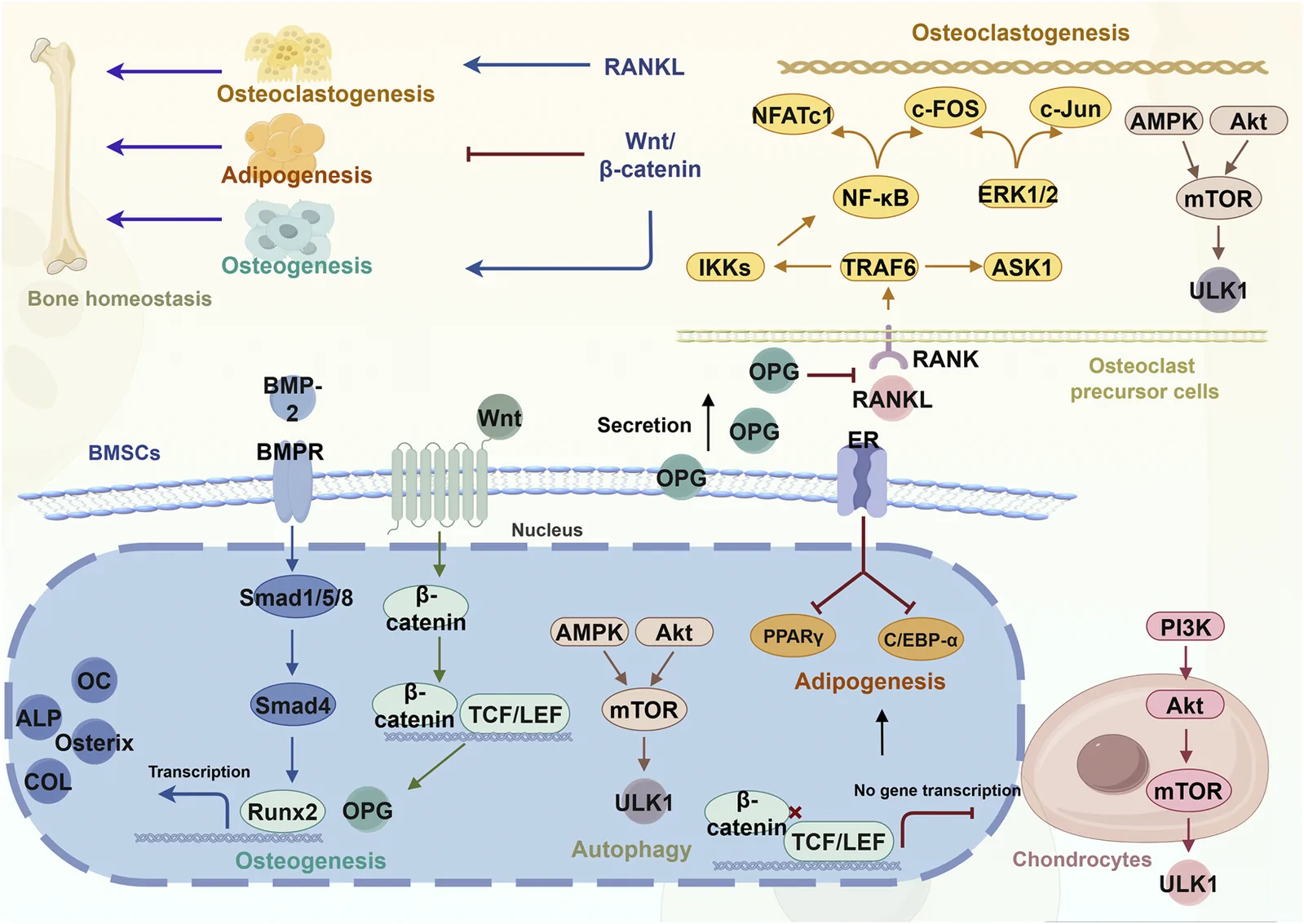
Signaling pathways modulated by ICA in bone diseases. Schematic overview of key pathways governing bone homeostasis, including osteogenesis (BMP/Smad, Wnt/β-catenin), and osteoclastogenesis (RANKL/RANK axis). mTOR signaling regulate autophagy in osteoblasts, osteoclasts, and chondrocytes. The cross (X) denotes separation.

While ICA can elevate BMD in ovariectomized rodents, caution is warranted when extrapolating such preclinical findings to human postmenopausal osteoporosis. The OVX rat model mimics acute estrogen deficiency accompanied by drastically elevated bone turnover. In contrast, human osteoporosis is a chronic age-related disorder with multifactorial pathogenesis, involving hormonal decline, deteriorated bone quality, muscle loss, persistent inflammation and cellular senescence (Cosgrove et al., 2014). Furthermore, most preclinical investigations adopt supraphysiological doses of ICA. Given its low oral bioavailability and plasma concentrations, such high drug exposure cannot be achieved in humans.

### 3.2 Amelioration of erectile dysfunction

As a representative kidney-Yang tonic in TCM, Epimedii Folium has been historically prescribed for erectile dysfunction (ED) and seminal emission. ICA underpins these effects across diverse ED models, including nerve injury-induced ED (BCNI), hypogonadism, and diabetes-related vasculogenic ED (see Table 4).

**TABLE 4:** Pharmacological effects of ICA in erectile dysfunction.

Subjects	Control groups	Dose/conc	Pharmacological effects	Key signaling pathways	Current limitations
BCNI rats (Xu Y. et al., 2017)	*In vivo*: (PC) Sildenafil	1.5 mg/kg/d	Producing a higher mean ICP/MAP ratio than sildenafil*	—	Conclusion mainly suitable for slight neurogenic erectile dysfunction
Castrated rats (Liu et al., 2005)	*In vivo*: (NC) Sham	1 mg/kg/d	Promoting erectile function (ICP/MAP* and percentage of smooth muscle*)	NOS signaling	The precise molecular mechanism linking ICA to NOS upregulation remains unexplored
T1DM rats (Yang et al., 2025)	*In vivo*: (NC) Ctrl	10 mg/kg/d	Promoting erectile function (ICPmax/MAP*); Reducing endothelial cell pyroptosis*, and ferroptosis*	—	Unexplored roles of necroptosis and PANoptosis in T1DM-induced cavernous tissue damage
SHR (Li X. et al., 2022)	*In vivo*: (NC) WKY rats	10 mg/kg/d	Improving erectile function, reducing endothelial microparticles, and platelet activation (ICPmax/MAP*, MAP**, PLT*, MPV*, and PDW*)	—	Lack of isotope labeling technique to verify ICA’s effect on EPC maturation
Rats treated with prostate radiation (Deng et al., 2022)	*In vivo*: (NC) Ctrl	10 mg/kg/d	Improving erectile function and inhibiting MAMs formation (ICPmax/MAP**)	eNOS signaling	The roles of other MAMs constituents remain uninvestigated.
12-month-old ICR mice (Sun et al., 2025)	*In vivo*: (NC) Ctrl	100 mg/kg/d	Protecting against testicular aging (sperm count**, testosterone levels** and SA-β-gal-positive area**)	ESR1/AKT	Lack of experimental validation for ICA–target binding
SHR (Li et al., 2025)	*In vivo*: (NC) WKY rats	10 mg/kg/d	Improving the erectile function (ICPmax/MAP**)	AKT/eNOS/NO	Lack of direct molecular mechanism validation, and long-term pharmacological effect confirmation
Cavernous smooth muscle cells treated with SNP (Ning et al., 2006)	*Ex vivo*: (PC) Zaprinast and SNP	0.75–1.1 μM; 100 and 200 μM.	Inhibiting PDE5A1, A2, and A3 with an IC50 value of 1.0, 0.75, and 1.1 M, respectively; outperforming the control in maintaining greater cGMP levels	cGMP signaling	Discordance between *in vitro* PDE5 IC_50_ values and cellular cGMP outcomes, with the underlying mechanism remaining unclear
SHR (Long et al., 2018)	*In vivo*: (NC) WKY rats	10 mg/kg/d	Increasing the ICPmax/MAP ratio*	Inhibiting uncoupling of eNOS	Absence of functional enzyme activity assays; the effect of ICA on coupling and uncoupling of nNOS and iNOS remains to be established
Isolated rabbit CC tissue treated with SNP (Jiang et al., 2006)	*Ex vivo*: (NC) Vehicle	50 μmol/L	Increasing cGMP concentration** and inhibiting PDE5A1 and PDE5A2 expression*	NO-cGMP	Absence of PDE5 protein/enzymatic activity assays
STZ induced T1DM mice (Lu et al., 2024)	*In vivo*: (NC) Vehicle	40 and 80 mg/kg/d	Alleviating testicular damage and promoting spermatogenesis (ZO-1**, Occludin*, Claudin-11*, testosterone production*, sperm count and mobility**)	AMPK/Nrf2; AMPK/NF-κB p65	Drug delivery systems should be developed; high-throughput sequencing and network pharmacology are need
STZ induced diabetic rats (Liu et al., 2011)	*In vivo*: (NC) Vehicle	1, 5 and 10 mg/kg/d	Increasing the ICP/MAP ratio*	TGFβ1/Smad2	Undefined direct link between ICA and TGFβ1/Smad2 pathway modulation

Abbreviations: NC, negative control; PC, positive control; MAMs, mitochondria-associated membranes; ESR1, estrogen receptor 1. *p < 0.05, and **p < 0.01.

Notably, ICA improved erectile function in scenarios where conventional PDE5 inhibitors fail, such as post-prostatectomy neuropathy (Nehra and Kulaksizoglu, 2002), without inducing visual disturbances or priapism (Gong et al., 2017). In BCNI rats, combination therapy with ICA and sildenafil produced superior intracavernous pressure/mean arterial pressure (ICP/MAP) ratios compared to monotherapy (Xu et al., 2017). In castrated rats, ICA significantly elevated mean ICP and increased smooth muscle content (Liu et al., 2005). In type 1 diabetes mellitus (T1DM) rats, ICA mitigated endothelial dysfunction by reducing apoptosis, pyroptosis, and ferroptosis (Yang et al., 2025). Furthermore, ICA improved hemodynamic parameters in spontaneously hypertensive rats (SHR), concomitantly inhibiting endothelial microparticle formation and platelet activation (Li X. et al., 2022). It also conferred radioprotection to penile cavernous tissue by maintaining nitric oxide (NO) concentrations and inhibiting mitochondrial-associated membranes (Deng et al., 2022). Beyond erectile function, ICA improved reproductive health in aging animal models by elevating testosterone levels and sperm counts (Sun et al., 2025). The underlying mechanisms involving the NO/cyclic guanosine monophosphate (cGMP) pathway and AMP-activated protein kinase (AMPK)/nuclear factor erythroid 2-related factor (Nrf2) signaling are detailed in Section 3.6.

### 3.3 Neuroprotective effects

TCM theory posits that “Kidney essence nourishes the brain marrow”; thus, Kidney deficiency leads to marrow depletion, manifesting as dementia and cognitive decline. Aligning with this, Epimedii Folium is prescribed to “tonify the Kidney and benefit the brain.” Accordingly, ICA’s therapeutic scope extends to the central nervous system, with emerging evidence supporting its efficacy against Alzheimer’s disease (AD), Parkinson’s disease (PD) stroke, and depression (see Table 5).

**TABLE 5:** Pharmacological effects of ICA in neurological disorders.

Indications	Subjects	Control groups	Dose/conc	Pharmacological effects	Key signaling pathways	Current limitations
AD (Zhang et al., 2014)	*In vivo*: Amyloid precursor protein transgenic mouse	(NC) WT mice	100 μM/kg	Improving cognitive deficits and reducing Aβ deposition (escape latency**, target quadrant search** and Aβ1-42 contents**)	—	BACE-1 activity was not directly assayed
Formaldehyde-induced injury (Song et al., 2016)	*Ex vivo*: SH-SY5Y cells	(NC) Vehicle	1 µM	Protecting cells from formaldehyde-induced cell death*	—	​
AD (Zhu et al., 2019)	*In vivo*: APP/PS1 mice	(NC) WT mice	60 mg/kg/d	Ameliorating cognitive deficits, Aβ deposition and inflammation (escape latency*, Aβ40*, insoluble Aβ42*, IL-1β*, IL-17A*, IL-12 p70*, and MCP-1*)	—	Lack of advanced single-cell and synaptic-level analyses
AD (Lu et al., 2025)	*In vivo*: Lateral ventricle injection of Aβ_1-42_	(PC) Donepezil	30 mg/kg/d	Improving memory impairment and neuronal damage (escape latency***, Nissl bodies** and TUNEL positive cells***)	cGAS	Incomplete mechanistic dissection of the cGAS-STING pathway and lack of assessment in non-microglial cell types
PD (Ma et al., 2023)	*In vivo*: A53T Tg mice; *Ex vivo*: SH-SY5Y cells	*In vivo*: (NC) WT mice; *Ex vivo*: (NC) vehicle	*In vivo*: 50 and 100 μM/kg; *Ex vivo*: 40 µM	Improving motor function and coordination (time on rotarod*, pole test performance score**)	—	Mechanistic evidence limited to changes in Parkin expression rather than direct target
Perimenopausal depression (Cao et al., 2019)	*In vivo*: Oophorectomy and chronic unpredictable stimulation	(NC) Sham	25 mg/kg/d	Improving depression and the pathological changes in the ovaries (stand-up time*, movement distance* and immobility time*)	PI3K-AKT	Absence of using pathway inhibitors in mechanistic studies
Spinal cord injury (Jia et al., 2019)	*In vivo*: Rats receiving weight-drop injury	(NC) Sham	35 μmol/kg	Promoting locomotor functional recovery (Beattie and Bresnahan score* and spinal cord water content*)	—	Lack of morphological identification of the specific cell types
Ischemia-related brain injury (Wang et al., 2009)	*Ex vivo*: Primary cortical neuron	(NC) Ctrl	1.2 µM	Enhancing neuronal viability*	MAPK/P38/SIRT1	Lack of *in vivo* corroboration
Ischemia-related brain injury (Zheng et al., 2008)	*In vivo*: Ischemia reperfusion-treated rats	(PC) Ergoloid mesylate	2.5, 5 and 10 mg/kg/d	ICA alone did not obviously improve cognitive impairment	—	Incomplete mechanistic characterization beyond oxidative stress and apoptosis pathways
AD (Jiang X. et al., 2019)	*In vivo*: Rats receiving intracerebro entricular injection of human Aβ_1-42_ peptide	(NC) Ctrl	30 mg/kg	Improving cognitive deficits and reducing Aβ (residence time*, latency time* and Aβ_42_*)	PI3K/AKT/mTOR	Poor blood-brain barrier penetration and incomplete mechanistic insight into its regulation of APP processing
Ischemia-related brain injury (Zhang et al., 2010)	*Ex vivo*: Primary cortical neurons	(NC) Ctrl	1.2 µM	Protecting against H_2_O_2_-induced neurotoxicity (cell viability*)	SIRT1	Lacking *in vivo* validation
Stroke (Zhu et al., 2010)	*In vivo*: middle cerebral artery occlusion mouse	(NC) Ctrl	100 mg/kg/d	Improving neurological scores, infarct size and brain edema (neurological scores* and brain water*)	SIRT1/PGC-1α	Incomplete characterization of upstream SIRT1 regulatory mechanisms.
AD (Liu J. et al., 2024)	*In vivo*: 3 × Tg-AD mice *Ex vivo*: HT22 cells injured by Ab25-35	*In vivo*: (NC) WT mice; *Ex vivo*: (NC) Ctrl	*In vivo*: 60 mg/kg/d *Ex vivo*: 10 µM	Improving cognitive function and autonomous behavior (escape latency*, locomotor distance* and nesting score***); reducing neuronal damage (LDH release*** and cell viability***)	Wnt/β-catenin	The discrepancy in PFKM1 regulation between animal and cellular models remaining mechanistically unresolved.
PD (Chen et al., 2017)	*In vivo*: 1-methyl-4-phenyl-1,2,3,6-tetrahydropyridine-induced mouse model	(NC) Ctrl	100 mg/kg/d	Ameliorating the decreased striatum DA content and the loss of tyrosine hydroxylase immunoreactive neurons (striatum DA content* and neuronal death*)	PI3K/Akt or MEK/ERK	Upstream signaling mechanisms of pathway activation remaining undefined
BD (Xiao et al., 2016)	Participants co-morbid BD and alcohol use disorder	None	300 mg/d for 8 weeks	Relieving depression and reducing alcohol consumption (HAMD* and heavy drinking days*)	—	Small open-label, single-arm pilot trial

Abbreviations: NC: negative control; PC: positive control; DA: dopamine; BD: bipolar disorder; HAMD: Hamilton Rating Scale for Depression. *p < 0.05, **p < 0.01, and ***p < 0.001.

In AD models, ICA ameliorated cognitive deficits by reducing β-amyloid deposition (Zhang et al., 2014), tau phosphorylation (Song et al., 2016) and neuroinflammation (Zhu et al., 2019). It protected SH-SY5Y cells from formaldehyde-induced injury death by inhibiting tau protein phosphorylation (Song et al., 2016). ICA also suppressed microglial hyperactivation and promoted M1-to-M2 phenotypic shift in amyloid-β (Aβ)_1–42_ injected AD models (Lu et al., 2025). In PD models, it decreased α-synuclein aggregation in SH-SY5Y cells *ex vivo* and enhanced motor function in A53T α-synuclein transgenic (A53TTg) mice (Ma et al., 2023). Besides, ICA alleviated depressive-like behaviors in perimenopausal rats by restoring neurotransmitter balance and sex hormone levels (Cao et al., 2019). ICA promoted locomotor functional recovery post-spinal cord injury by attenuating oxidative stress, apoptosis, and inflammation (Jia et al., 2019). ICA could enhance neuronal viability following oxygen and glucose deprivation *ex vivo* (Wang et al., 2009). Clinically, an uncontrolled open-label study showed that 300 mg/day ICA for 8 weeks relieved depression and reduced alcohol consumption in comorbid patients (Xiao et al., 2016). The crosstalk between sirtuin 1 (SIRT1) and the cyclic GMP-AMP synthase (cGAS)/stimulator of interferon genes (STING) pathway in mediating these effects is analyzed in Section 3.6 (see Figure 4).

**FIGURE 4:**
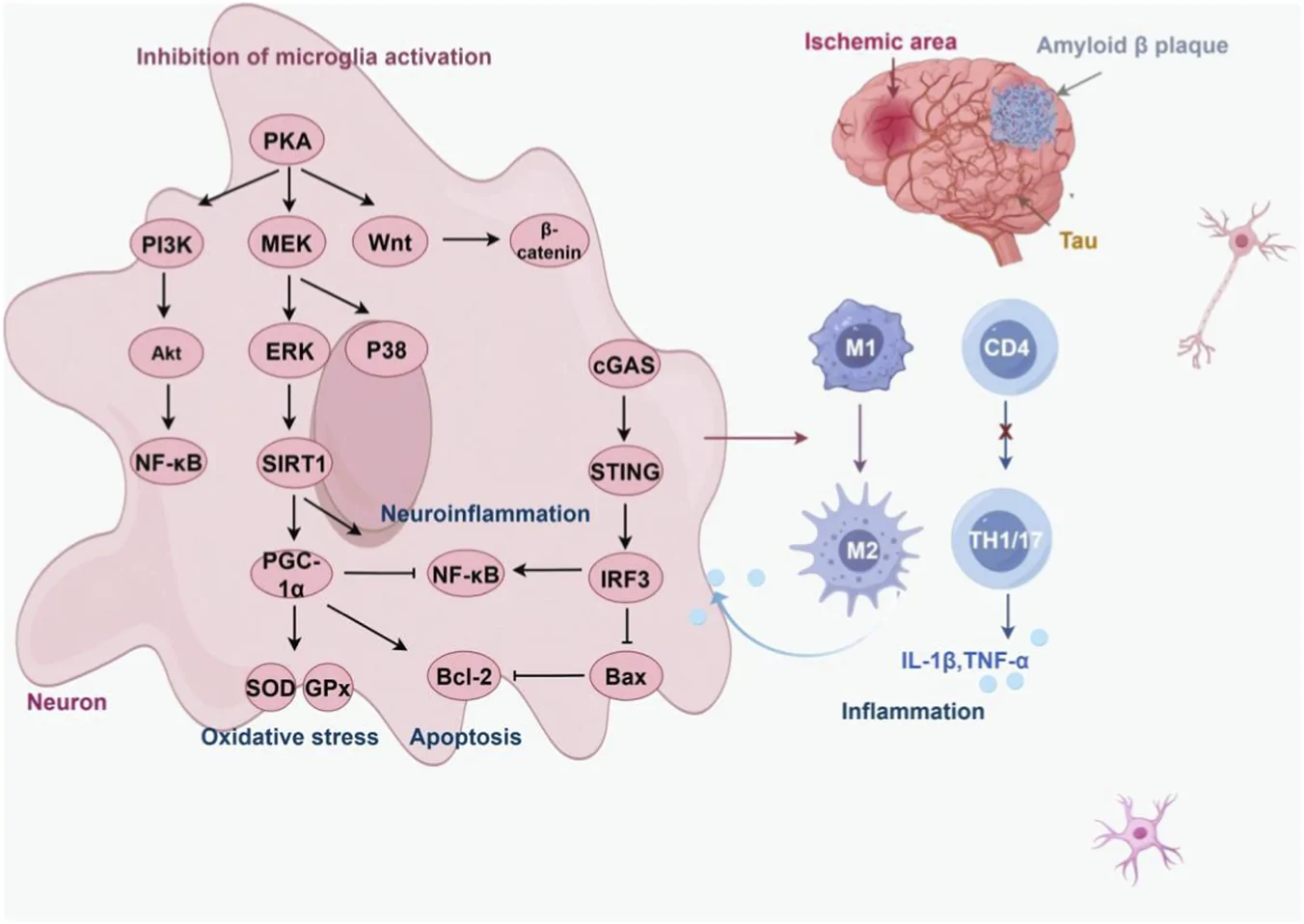
Signaling pathways modulated by ICA in neurological disorders. Overview of key signaling hubs governed by ICA in neurological disorders. Bidirectional interactions among neurons, macrophages, and CD4^+^ T cells underlie neuroinflammatory responses. Crosstalk between cGAS–STING and SIRT1/PGC-1α orchestrates NF-κB–driven neuroinflammation, Bax/Bcl-2–controlled apoptosis and PI3K/Akt survival signaling. The cross (X) denotes inhibition.

The neuroprotective effects of ICA are demonstrably context-dependent. For instance, while 10 mg/kg ICA failed to ameliorate cognitive deficits in rats subjected to cerebral ischemia/reperfusion injury (Zheng et al., 2008), it conferred significant benefits in chronic neurodegenerative models (Linkermann et al., 2013) This discrepancy likely stems from acute blood–brain barrier disruption, which alters drug distribution and bioavailability. Given ICA’s low oral bioavailability, the 10 mg/kg regimen likely results in sub-therapeutic systemic exposure, precluding meaningful brain accumulation. Effective neuroprotection is consistently reported at doses of 30–100 mg/kg, highlighting a steep dose–response relationship. Taken together, these findings indicate that ICA’s neuroprotective efficacy is contingent upon specific pathological stages and pharmacologically optimized dosing regimens. Furthermore, these preclinical findings predominantly derive from rodent models of acute injury or transgenic neurodegeneration, which inadequately recapitulate the chronic, progressive pathophysiology of human neurodegenerative diseases.

### 3.4 Emerging role in anti-fibrosis

The traditional indication of Epimedii Folium for dispelling wind-dampness aligns conceptually with its modern application in chronic fibrosis disorders. By suppressing excessive extracellular matrix deposition, ICA offers a pharmacological rationale for its historical use in rheumatic and degenerative diseases. Accumulating evidence has demonstrated ICA’s robust antifibrotic efficacy across multiple organs, including the lung, liver, kidney and heart (see Table 6).

**TABLE 6:** Pharmacological effects of ICA in fibrotic diseases.

Indications	Subjects	Ctrl groups	Dose/conc	Pharmacological effects	Key signaling pathways	Current limitations
Pulmonary fibrosis (Du et al., 2022)	*In vivo*: BLM-treated rats	(NC) Vehicle (PC) Pirfenidone	60 mg/kg/d	Improving lung histoarchitecture	CB signaling	Lack of CB2-specific causal validation. The pharmacological effects of ICA has yet to be validated in human IPF patients
NASH (Choi et al., 2023)	*In vivo*: MCD diet treated mice	(NC) Methionine choline sufficient diet mice	50 and 100 mg/kg/d	Inhibiting hepatic steatosis and fibrosis (serum ALT*, serum AST*, hepatic triglyceride*, NASH score*, and fibrosis area*)	Nrf2-xCT/GPX4	Absence of a direct ferroptosis inhibitor control group
Liver fibrosis (Ye et al., 2020)	*In vivo*: CCL_4_- treated mice; *Ex vivo*: Mouse primary HSCs	*In vivo*: (NC) Olive oil plus oral PBS *Ex vivo*: (NC) DMSO	*In vivo*: 100 mg/kg/d; *Ex vivo*: 10 μM	Inhibiting liver fibrosis (Collagen expression** and αSMA levels**); inhibiting EMT and fibrosis property of HSCs (the expression of αSMA** and Col1A1**, cell proliferation*, and cell migration**)	Hedgehog signaling	The upstream p53 regulatory mechanism left unexplored
Liver fibrosis (Algandaby et al., 2017)	*In vivo*: Thioacetamide-treated rats	(NC) Saline and DMSO	50 mg/kg/d	Improving liver dysfunction and fibrosis (ALT*, AST*, Albumin*, Col-1a* and hydroxylproline*)	mTOR/Beclin-1	Absence of mTOR inhibitors or activators to elucidate the mechanisms
NS (Duan et al., 2024)	*In vivo*: Doxorubicin treated rats	(NC) Vehicle (PC) prednisone	50 mg/kg/d	Improving renal function and fibrosis (ALB**, BUN**, Scr**, collagen deposition**)	—	Lack of NLRP3 knockout mechanistic validation and insufficient safety data
Renal fibrosis (Chen H. A. et al., 2019)	*In vivo*: UUO mice	(NC) Sham (PC) Candesartan	20 mg/kg/d	Antifibrotic and anti-inflammatory effects (collagen deposition*, TGF-β*, CTGF*, α-SMA* and FN*)	—	Incomplete mechanistic dissection of ICA metabolites and SIRT-1 involvement
Renal fibrosis (Wang et al., 2021)	*In vivo*: 5/6 (A/I)rats *Ex vivo*: NRK-49F cells	*In vivo*: (NC) sham *Ex vivo*: (NC) DMSO	*In vivo*: 40 mg/kg/d *Ex vivo*: 5 μM	Improving kidney injury and fibrosis (Scr***, BUN***, FN***, α-SMA***, CTGF**, and PCNA**); Suppressing the IL-1β-induced activation of renal fibroblasts (FN*, α-SMA***, CTGF*, and PCNA***)	IL-1β/TGF-β	Focus on renal fibroblasts without assessing other renal cell types, and the absence of a positive control
Diabetic nephropathy (Wang et al., 2024)	*In vivo*: STZ treated rats; *Ex vivo*: High glucose treated HK-2 cells	*In vivo*: (NC) Water *Ex vivo*: (NC) Vehicle	*In vivo*: 80 mg/kg/d; *Ex vivo*: 2 μM	Alleviating renal fibrosis (BMP6****, α-SMA**** and CTGF**) and HK-2 cell fibrosis (Collagen I**, α-SMA**)	BMP6/Smad	Without exploring other potential ICA targets
Pulmonary fibrosis (Deng et al., 2023)	*In vivo*: BLM-treated mice; *Ex vivo*: NIH-3T3 cells	*In vivo*: (NC)Vehicle *Ex vivo*: (NC)DMSO	*In vivo*: 10 mg/kg/d ICA II; *Ex vivo*: 5 μM ICA II	Improving lung inflammation and fibrosis (inflammation score*, fibrosis score **); Inhibiting TGF-β1-mediated fibrogenesis (Collagen I***, FN*** and α-SMA***)	WNT/β-catenin	Absence of WNT inhibitors or activators to elucidate the mechanisms
Renal fibrosis (Jia et al., 2021)	*In vivo*: T2DM rats *Ex vivo*: HK-2 and NRK-49F cells	*In vivo*: (NC) Saline *Ex vivo*: (NC) Vehicle	*In vivo*: 80 mg/kg/d *Ex vivo*: 10 μM	Alleviating renal function and fibrosis (24 h urinary volume**, creatinine clearance*, collagen area**, LC3*, p62**, and α-SMA****); Inducing cell autophagy (BECN1***, SQSTM1****, COL1A1****, ACTA2****, and FN1****)	Androgen Receptor/mTOR	Absence of biophysical validation for ICA-target interactions
Renal fibrosis (Ding et al., 2024)	*In vivo*: UUO mice; *Ex vivo*: TGF-β1-exposed HK-2 cells	*In vivo*: (NC) Sham *Ex vivo*: (NC) Vehicle	*In vivo*: 50 mg/kg/d *Ex vivo*: 50 μМ	Improving renal function and pathological lesions (Serum BUN* and Cr*, collagen I* and α-SMA*); Attenuating pro-inflammatory and fibrotic phenotype (collagen I**, α-SMA**, IL-1β*, TNF-α** and IL-6**)	Nrf2/HO-1	Upstream mechanisms of ICA-driven Nrf2 activation remains unclear
CTIN (Hou et al., 2022)	*In vivo*: cyclosporine A and ochratoxin A treated rats *Ex vivo*: TGF-β1-exposed HK-2 cells	*In vivo*: (PC) SeMet, (NC) Vehicle. *Ex vivo*: (PC) SeMet, (NC) Vehicle.	*In vivo*: 20 mg/kg/d *Ex vivo*: 0–32 μg/mL	Improving renal function and fibrosis (kidney index*, UP*, BUN**, Scr*, collagen I*, and α-SMA**); Attenuating fibrotic phenotype (collagen I* and TGF-β1*)	TLR4/NFκB	*In vivo* metabolic crosstalk between ICA and SeMet together with their pharmacokinetics remains unassessed
Myocardial infarction (Jia et al., 2023)	*In vivo*: Myocardial infarction rat *Ex vivo*: RAW264.7	*In vivo*: (NC) Sham. *Ex vivo*: (NC) Vehicle.	*In vivo*: 30, 60 and 120 mg/kg/d *Ex vivo*: 60 μM	Improving left ventricular remodeling and myocardial fibrosis; Inhibiting fibrotic phenotype (TGF-β****, p-Smad2**** and p-Smad3****).	TGF-β1/Smad	Short follow-up in a rat-only model without dose optimization, and long-term safety data
Pulmonary fibrosis (Du et al., 2021)	*In vivo*: BLM-treated rats *Ex vivo*: NIH/3T3 cells	*In vivo*: (PC) Pirfenidone *Ex vivo*: (NC) DMSO	*In vivo*: 60 mg/kg/d *Ex vivo*: 50 and 100 μM	Reducing the collagen deposition and inflammation (collagen area*, the ratio of type I/III**, TGF-β* and IL-1β*); Inhibiting the expression of profibrogenic genes (collagen I* and FN*)	Hippo pathway	BLM induced models with limited translatability to human IPF
Renal fibrosis (Yao et al., 2024)	*In vivo*: STZ inducedT2DM mice	(PC) Met	80 mg/kg/d	Inhibiting the renal fibrosis by suppressing EMT (CD31*, VE-cad**, FN**, α-SMA* and FSP-1*)	AR/RKIP	Male-only animal models limit the generalizability of findings to female populations
Renal fibrosis (Zhang et al., 2022)	*In vivo*: UUO mice; *Ex vivo*: TGF-β1-stimulated HK-2 cells	*In vivo*: (NC) Vehicle *Ex vivo*: (NC) Vehicle	*In vivo*: 60 mg/kg/d; *Ex vivo*: 50 µM	Attenuating tubular injury and renal fibrosis (tubular injury scores*, Masson-positive area* and Sirius Red positive area*); Decreasing pro-fibrotic phenotype (FN*, type I collagen* and α-SMA*)	Notch2/Hes-1	Lacking causal pathway validation

Abbreviations: NC, negative control; PC, positive control; IPF, idiopathic pulmonary fibrosis; HSCs, hepatic stellate cells; MCD, Methionine choline-deficient; xCT, cystine/glutamate transporter; GPX4, glutathione peroxidase 4; SeMet, Selenomethionine; VE-cad, Vascular endothelial cadherin; AR, androgen receptor; RKIP, RAF, kinase inhibitor. *p < 0.05, **p < 0.01, ***p < 0.001, and ****p < 0.0001.

ICA attenuated myocardial fibrosis and improved cardiac performance in type 2 diabetes mellitus (T2DM) rats (Zhang L. et al., 2021). In bleomycin (BLM)-induced pulmonary fibrosis, ICA inhibited epithelial–mesenchymal transition (EMT) and preserved lung histoarchitecture (Du et al., 2022). In methionine choline-deficient diet-induced non-alcoholic steatohepatitis (NASH) models, ICA mitigated inflammation, steatosis, hepatocyte ballooning, and fibrosis (Choi et al., 2023). Similarly, ICA attenuated EMT and collagen deposition in livers from CCl_4_-challenged mice (Ye et al., 2020), and diminished angiogenesis- and autophagy-mediated collagen accumulation in thioacetamide-treated mice (Algandaby et al., 2017). Renal fibrosis was also ameliorated by ICA across diverse etiologies, including diabetic nephropathy, nephrotic syndrome (NS), unilateral ureteral obstruction (UUO), and 5/6 nephrectomy with infarction-5/6 (A/I) model. Specifically, ICA improved renal function by inhibiting pyroptosis and EMT in doxorubicin induced NS mice (Duan et al., 2024), reduced collagen deposition and profibrotic factor expression in UUO rats (Chen H. A. et al., 2019), and attenuated cytokine release and histopathological injury in 5/6 (A/I) rats (Wang et al., 2021). *In vitro*, ICA inhibited the expression of α-SMA, fibronectin (FN), and connective tissue growth factor (CTGF), and type I collagen in HK-2 cells (Wang et al., 2024). Notably, its primary metabolite, ICA II, could also decrease collagen deposition and the expression of IL-1β, TNF-α, TGF-β1 and PDGF (Deng et al., 2023). Mechanistically, the TGF-β/Smad, BMP6/Smad, and Wnt/β-catenin cascade exhibit complex interplay and mutual regulation, as elaborated in Section 3.6 (see Figure 5).

**FIGURE 5:**
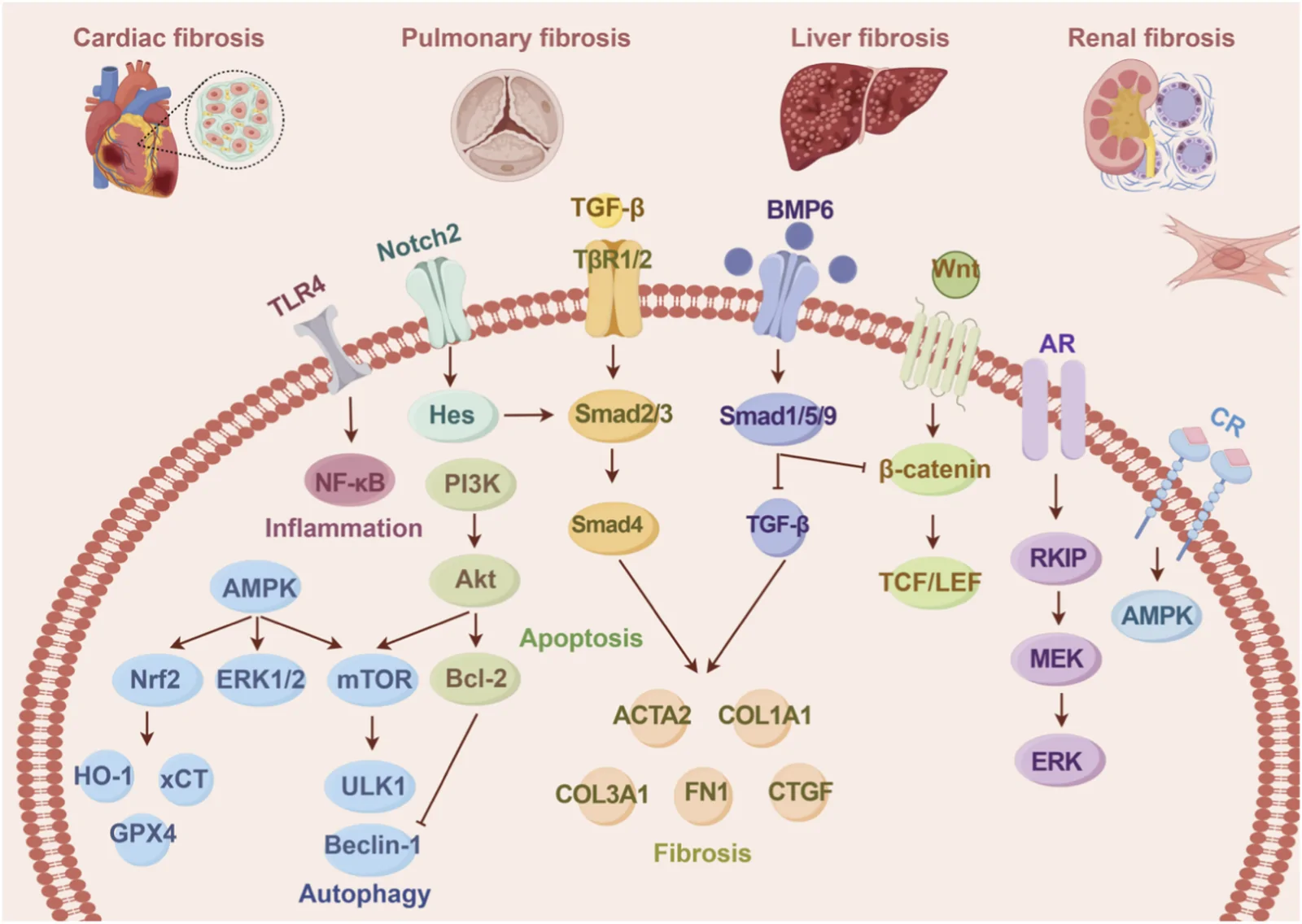
Signaling pathways modulated by ICA in fibrotic diseases. Schematic illustrating core signaling axes modulated by ICA that govern cardiac, pulmonary, hepatic, and renal fibrosis. Key cascades include TGF-β/Smad, BMP/Smad, Wnt/β-catenin, PI3K/Akt/mTOR, AMPK/Nrf2, and AR/MEK/ERK, which coordinately regulate fibrotic gene transcription, redox homeostasis, autophagy, and cell survival. Arrows (→) denote activation; blunt-ended lines (⊣) denote inhibition.

While ICA effectively attenuates fibrosis in acute models, such as BLM-induced lung injury and CCl_4_-induced liver injury, the clinical translatability of these findings is critically limited. These rodent models typically induce tissue damage over weeks, whereas human fibrotic diseases, such as idiopathic pulmonary fibrosis (IPF) and NASH, evolve insidiously over decades (Richeldi et al., 2017; Schuster et al., 2018). Consequently, therapeutic effects observed in short-term animal studies cannot reliably predict clinical outcomes in patients with chronic, complex fibrotic disorders. Furthermore, significant interspecies PK disparities also represent a major translational barrier. Thus, long-term investigations are required to define optimal therapeutic dosages for human use.

### 3.5 Anti-cancer potential

Although not a classical TCM indication, the anticancer potential of ICA exemplifies how traditional botanicals may serve as promising adjuvants in modern oncology. ICA exhibits broad-spectrum antitumor effects across various malignancies, including hepatocellular carcinoma (HCC), breast, ovarian, cervical and colorectal cancers (see Table 7).

**TABLE 7:** Pharmacological effects of ICA in cancers.

Indications	Subjects	Ctrl groups	Dose/conc	Pharmacological effects	Key signaling pathways	Current limitations
HCC (Li et al., 2014)	*In vivo*: Murine HCC cancer xenograft model; *Ex vivo*: HepG2 and SMMC7721 cells	*In vivo*: (NC) Saline *Ex vivo*: (NC) DMSO	*In vivo*: 40 mg/kg/d; *Ex vivo*: 10 μM	Reducing tumor volume*; Sensitizing cells to apoptosis*	NF-κB signaling	The concrete pharmacokinetic features of ICA–arsenic trioxide interaction remain uncharacterized, and no testing was conducted in clinically relevant orthotopic liver tumor models
HCC (Gao et al., 2025)	*In vivo*: Murine HCC cancer xenograft model; *Ex vivo*: Bel-7402 and HepG2 cells	*In vivo*: (NC) Ctrl *Ex vivo*: (NC) DMSO	*In vivo*: 40 mg/kg/d; *Ex vivo*: 20 μM	Suppressing the tumor growth (tumor volume* and tumor weight**); Inhibiting HepG2 proliferation** and invasion**	PI3K/AKT/mTOR	Use of HepG2 cells may not fully reflect malignant HCC features
HCC (Fan et al., 2019)	Clinical participants: Advanced HCC patients with Child-Pugh Class A or B; *Ex vivo*: Myeloid cells	Clinical studies: ; *Ex vivo*: (NC) DMSO (PC) LPS	Clinical studies: 600 or 800 mg/d ICT; *Ex vivo*: 2, 2.5 or 10 μM ICT	46.7% of patients achieved clinical benefit; Blocking the immune-suppression in myeloid cells (M-CSFR expression*).	IL-6/Jak2/Stat3	Small sample size and no positive control group in clinical trials
HCC (Shao et al., 2024)	Clinical participants: HCC patients with unresectable IIA-IIIB	(PC) ICT monotherapy	ICT monotherapy or combined with PD-1/L1 and/or anti-VEGF therapy	Combination treatment obtained longer PFS (8.94 months vs. 5.52 months), while the mono-group attained a higher ORR (12.50% vs. 6.06%)	—	Discrepancy between ORR and PFS outcomes
HCC (He et al., 2026)	Clinical participants: HCC patients	None	600 mg twice daily ICT	Combination therapies yielded superior DCR (93.8% vs. 60%), PFS (5.7 months vs. 3.5 months), and OS (10.6 months vs. 9.4 months)	—	Single-center design and relatively small sample size
Ovarian cancer (Li et al., 2015)	*Ex vivo*: A2780 cells	(NC) DMSO	25 μM	Suppressing cell proliferation**, and increasing apoptosis**	None	Incomplete elucidation of the upstream molecular mechanisms by which ICA regulates the miR-21/PTEN/RECK/Bcl-2 axis
Ovarian cancer (Jiang S. et al., 2019)	*Ex vivo*: SKVCR cells	(NC) DMSO	20 μg/mL	Inhibiting cell viability*** and autophagy*, promoting apoptosis***	AKT/mTOR/ATG5	Lack of validation in other ovarian cancer cell models, and *in vivo* models
Prostate cancer (Chen C. et al., 2023)	*In vivo*: Mouse RM1-Luc PCa bone metastasis model; *Ex vivo*: Raw264.7 cells or BMMs	*In vivo*: (NC) Saline *Ex vivo*: (NC) Vehicle	*In vivo*: 20 and 40 mg/kg/d; *Ex vivo*: 20 µM	Inhibiting cancer bone metastases and destruction (tumor growth** and TRAP-positive areas*); Inhibiting osteoclast differentiation.	TAM/CCL5	Whether other bone TME components mediate ICA’s pharmacological effects remains to be elucidated
Breast cancer (Song et al., 2020)	*In vivo*: MDA-MB-231 tumor-bearing mice, 4T1 tumor-bearing mice and 4T1 cells xenograft pulmonary metastasis model; *Ex vivo*: MDA-MB-231, MDA-MB-453, 4T1, and MCF-10A	*In vivo*: (NC) Ctrl *Ex vivo*: (NC) Vehicle	*In vivo*: 20 mg/kg/d; *Ex vivo*: 10 μM	Inhibiting tumor growth and pulmonary metastases (tumor volume* tumor weight*, and number of lung nodules**); Selectively inhibiting breast cancer cell proliferation *in vitro* (cell viability*** and colony formation***)	SIRT6/NF-κB	Upstream mechanism of ICA-induced SIRT6 upregulation is unexplored
Colorectal cancer (Chen Y. et al., 2025)	*In vivo*: AOM/DSS-induced CRC model and CT26-WT syngeneic subcutaneous tumor model; *Ex vivo*: CT26-WT, HCT116 cells, RAW264.7, and THP-1 cells	*In vivo*: (PC) 5-FU *Ex vivo*: (NC) Vehicle	*In vivo*: 100 mg/kg/d; *Ex vivo*: 40 μM	Attenuating tumor growth (tumor number**** in BALB/c mice; tumor volume* and tumor weight** in CT26-WT syngeneic subcutaneous tumor model); Inhibiting the proliferation of CT26- WT**** and HCT116**	PI3K/AKT	Lack of causal validation of the PI3K/AKT-mediated mechanism
Breast cancer (Ma et al., 2014)	*Ex vivo*: SKBr3 cells	(NC) Vehicle	1 × 10^−7^ M ICA or ICT	The maximal cell proliferative effects were 148% and 144% at a dose of 1 × 10^−7^ M ICA or ICT	EGFR-MAPK	Lacking validation in other breast cancer cell models, and *in vivo* models
Breast cancer (Zhao et al., 2024)	*In vivo*: TNBC 4T1 xenograft mouse model; *Ex vivo*: MDA-MB-468, MDA-MB-231, and 4T1 cell	*In vivo*: (NC) Vehicle *Ex vivo*: (NC) Vehicle	*In vivo*: 20 mg/kg/d; *Ex vivo*: 12.5 µM	Inhibiting TNBC growth (tumor weight** and tumor volume**); Suppressing the viability**, proliferation***, migration***, and invasion***	AMPK/mTOR/ULK1	The study did not investigate the pharmacokinetics and pharmacodynamics of ICA
Cervical cancer (Li et al., 2021)	*In vivo*: U14 tumor-bearing mice; *Ex vivo*: SiHa cells	*In vivo*: (PC) Cyclophosphamide; *Ex vivo*: (NC) Vehicle	*In vivo*: 10, 20, 40 mg/kg/d; *Ex vivo*: 10, 30, 90, 120 and 150 μM	Inhibiting the growth of tumor (tumor weight** and tumor volume**); Inhibiting cell migration*, invasion*, and promoted the apoptosis** of SiHa cells	TLR4/MyD88/NF-κB and Wnt/β-catenin	No flow cytometry-based immune cell validation and unexplored TLR4 function in ICA’s anti-cervical cancer effects
Ovarian cancer (Fu et al., 2022)	*In vivo*: SKOV-3 cells were implanted into nude mice *Ex vivo*: SKOV-3 cells	*In vivo*: (PC) Cisplatin *Ex vivo*: (NC) Vehicle	*In vivo*: 20, 40, and 80 mg/kg; *Ex vivo*: 25, 50, 100, and 200 μM	Suppressing tumor growth; Suppressing cell proliferation (IC50 = 56.3 μM)	Wnt/β-catenin pathway	Lacking broader cellular validation
Ovarian cancer (Wang et al., 2020)	*Ex vivo*: SKOV3 cell	(NC) Vehicle	25, 50 and 100 μM	Promoting apoptosis** and inhibiting cell invasiveness**	PI3K/Akt	In vitro-only evidence from a single cancer cell line without *in vivo* corroboration
Ovarian cancer (Huang et al., 2019)	*Ex vivo*: HeLa cells	(NC) Vehicle	10, 20 and 40 μM	The IC50 of ICA was 20 μM	mTOR/PI3K/AKT	Warranting *in vivo* evaluation

Abbreviations: NC, negative control; PC, positive control; ORR, objective response rate; PFS, progression-free survival; DCR, disease control rate; OS, overall survival; BMMs, bone marrow-derived macrophages. *p < 0.05, **p < 0.01, and ***p < 0.001.

ICA significantly reduced tumor volume in murine HCC xenograft models and sensitized SMMC7721 and HepG2 Cells to apoptosis (Li et al., 2014). Its primary antitumor mechanisms involved inducing G_2_/M phase arrest and programmed cell death (Li et al., 2015; Jiang S. et al., 2019). Furthermore, ICA mitigated bone metastasis in prostate cancer by remodeling the immunosuppressive tumor microenvironment (Chen C. et al., 2023). In triple-negative breast cancer (TNBC) mouse models, ICA markedly suppressed tumor growth and pulmonary metastases by promoting CD4^+^ and CD8^+^ T cells infiltration (Song et al., 2020). In the AOM/DSS-induced colorectal carcinogenesis models, ICA reduced tumor numbers and increased colon length by suppressing M2 macrophage polarization (Chen Y. et al., 2025). Notably, ICA attenuated cisplatin-induced cytotoxicity in HEK-293 cells by suppressing oxidative stress, inflammatory responses, and apoptosis (Zhou et al., 2019), underscoring its supportive therapeutic potential. The antitumor effects of its metabolites, ICA II and ICT, are also well-documented. ICA II exhibited superior antiproliferative activity against HeLa cells compared to 5-fluorouracil (5-Fu), with IC_50_ values of 10 μM and 31.1 μM, respectively (Wu et al., 2012). ICT sensitized colorectal tumors to low-temperature photothermal therapy by inhibiting autophagy and inducing apoptosis (He et al., 2025), and restrains urothelial cancer progression by suppressing neutrophil infiltration and neutrophil extracellular trap formation (Mou et al., 2024). The dual modulation of the PI3K/Akt and AMPK/mTOR signaling axes by ICA in tumorigenesis is comprehensively discussed in Section 3.6.

Crucially, ICA functions not as a broad-spectrum cytotoxic agent but as a targeted adjuvant candidate for specific molecular subtypes. While ICA effectively suppressed cell proliferation in TNBC models, it exhibited minimal activity and may even promote proliferation in ER-positive subtypes such as SKBr3 cells (Ma et al., 2014). This underscores that precise patient stratification based on molecular profiling is an indispensable prerequisite for ICA’s clinical translation. Unlike conventional chemotherapeutics, ICA demonstrated selective cytotoxicity, with minimal toxicity toward normal cells, including mouse hepatocytes (Li et al., 2014), breast epithelial cells (Song et al., 2020) and HCvEpC cells (Huang et al., 2019), which underscored its favorable safety profiles for clinical translation.

### 3.6 Overview of integrated signaling pathways

ICA exerts pleiotropic pharmacological effects including bone anabolism, erectile dysfunction improvement, and neuroprotection, through the modulation of interconnected signaling networks such as AMPK/mTOR, ER signaling, eNOS/cGMP, and Nrf2/NF-κB (see Figure 6). Deciphering these cascades clarifies ICA’s multi-target therapeutic profiles, aligning with the TCM concept of “multi-system regulation.”

**FIGURE 6:**
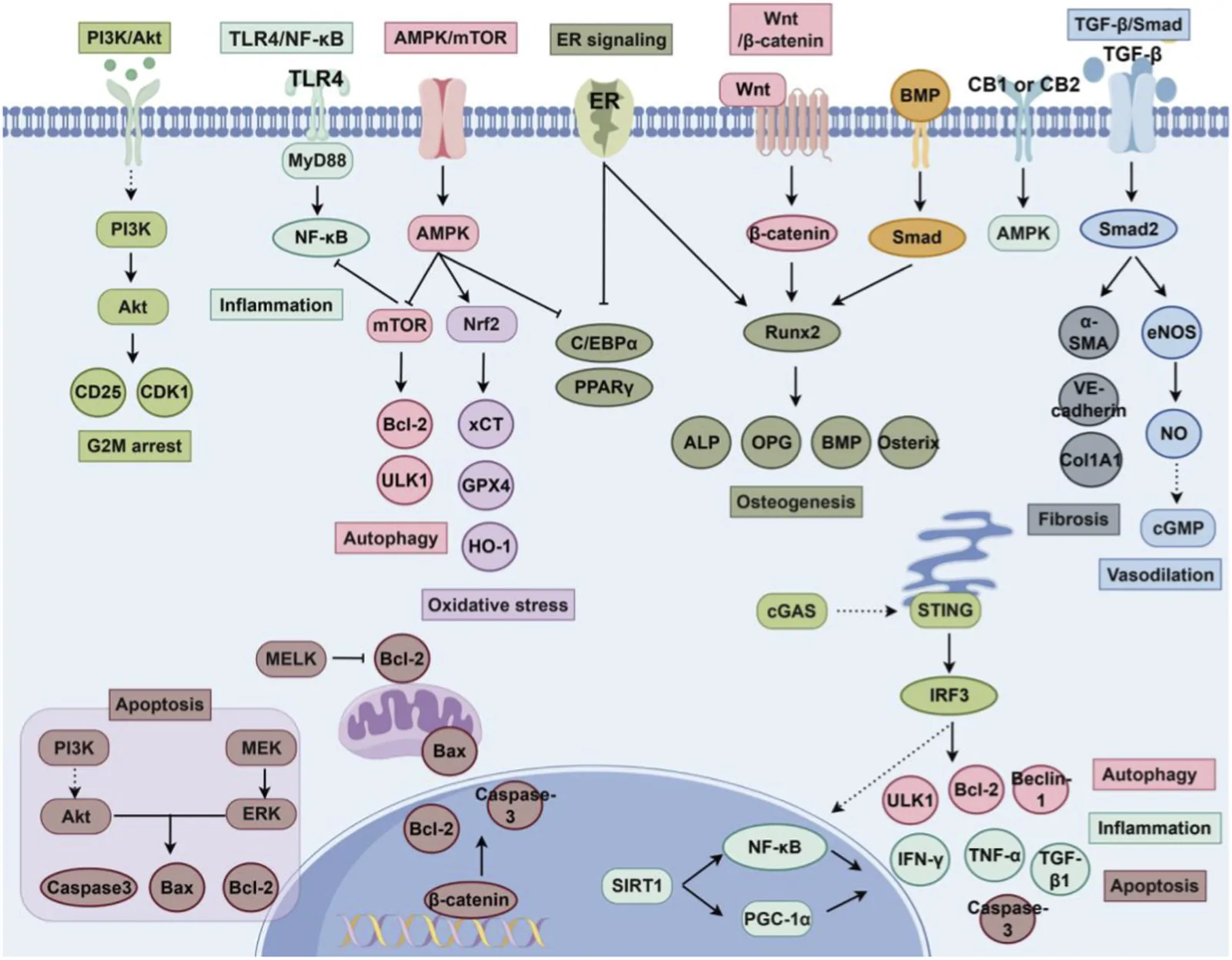
Multitarget regulatory landscape of ICA across disease contexts. Schematic overview of ICA orchestrating key biological processes—including osteogenesis, osteoclastogenesis, anti-inflammatory responses, anti-fibrosis, cell-cycle arrest, apoptosis, autophagy, and oxidative stress—primarily through canonical pathways such as ER signaling, Wnt/β-catenin, AMPK/mTOR, TLR4/NF-κB, TGF-β/Smad, and cGAS–STING.

#### 3.6.1 The AMPK/mTOR axis (low evidence)

ICA orchestrates cellular energy homeostasis primarily via AMPK activation and mTOR inhibition, thereby restoring autophagic flux (Zou et al., 2024). In bone marrow mesenchymal stem cells (BMSCs) from OVX rats, ICA regulated the balance between osteogenic and adipogenic differentiation through mTOR signaling (Zeng et al., 2026). This axis also facilitates the clearance of misfolded proteins and damaged organelles. In HK-2 cells, ICA induced autophagy by downregulating phosphorylated mTOR, thereby suppressing the expression of profibrotic markers (Jia et al., 2021). Notably, ICA’s effects are context-dependent in oncology: AMPK activation triggered autophagic cell death in aggressive MDA-MB-231 and U14 cells (Zhao et al., 2024), whereas it suppressed autophagy and enhanced chemosensitivity in SKVCR cells.

#### 3.6.2 ER signaling (preliminary evidence)

Engagement of ER signaling may mediate the osteoprotective effects of ICA. ICA bound to ERα, inducing rapid phosphorylation of ERK1/2 and Akt in UMR-106 cells (Ho et al., 2018). Functionally, it upregulated osteogenic markers (RUNX2, Col-1, BMP-2) while suppressing adipogenic markers (PPARγ, C/EBPα) in rat BMSCs via ER-dependent pathways (Li et al., 2018). ICA increased cell viability and ALP activity by activating the BMP-2/Smad5/Runx2 axis in rBMSC (Zhang X. et al., 2021; Zhang X. Y. et al., 2021). ICA also elevated the OPG/RANKL ratio through ligand-independent ER activation in UMR-106 cells, favoring bone formation (Xiao et al., 2014). Recent evidence suggested ICA promoted osteogenesis via epigenetic mechanisms by repressing SOST promoter methylation and increasing ERα binding (Chen X. et al., 2025).

#### 3.6.3 NOS/cGMP (low evidence)

ICA ameliorates erectile dysfunction predominantly through the eNOS/cGMP cascade. It elevated nitric oxide (NO) concentrations and upregulates neuronal nitric oxide synthase (nNOS), eNOS, and p-eNOS in irradiated penile tissues (Deng et al., 2022). ICA activated the AKT/eNOS/NO pathway while suppressing GRK2, thereby reducing oxidative stress in SHR (Li et al., 2025). Chronic treatment upregulated nNOS expression and increased trabecular smooth muscle content in castrated animal models (Long et al., 2018). *Ex vivo*, ICA elevated cGMP levels in rodent cavernous smooth muscle cells (Ning et al., 2006), without affecting cAMP in isolated rabbit CC tissues (Jiang et al., 2006). Inhibitors of NOS, sGC, and cGMP abolished the osteogenic effect of ICA on rBMSCs (Zhai et al., 2014).

#### 3.6.4 Nrf2 and NF-κB (low evidence)

ICA mitigates oxidative stress and inflammation via dual regulation of Nrf2 and NF-κB. In diabetic mice, ICA protected testicular integrity and spermatogenesis via AMPK-mediated Nrf2 activation and NF-κB inhibition (Lu et al., 2024). It stabilizes Nrf2, inducing HO-1 expression and glutathione synthesis. Nrf2 inhibition abolished ICA’s protection against mitochondrial dysfunction and renal fibrosis (Ding et al., 2024). Concurrently, ICA suppressed NF-κB and NLRP3 inflammasome activation, reducing pro-inflammatory cytokines production. ICA inhibited osteoclast differentiation and pro-inflammatory cytokine synthesis by modulating mitogen-activated protein kinase (MAPK)/NF-κB signaling (Hsieh et al., 2011) and downregulating c-Fos and nuclear factor of activated T cells c1 (NFATc1) (Kim et al., 2018). It suppressed the tumor growth in U14 cervical carcinoma xenograft mice via inhibition of the TLR4/MyD88/NF-κB signaling pathway (Li et al., 2021).

#### 3.6.5 TGF-β/Smad & endocannabinoid (low evidence)

Functional crosstalk between TGF-β and endocannabinoid axes plays significant roles in fibrotic progression. In models of diabetic erectile dysfunction, ICA preserved penile hemodynamics and endothelial integrity by inhibiting TGF-β1/Smad2 cascade (Liu et al., 2011). Concordantly, ICA attenuated renal fibrosis in chronic tubulointerstitial models by downregulating collagen and α-SMA expression via targeted inhibition of TGF-β1/Smad2 axis (Hou et al., 2022). In myocardial infarction models, it reduced collagen deposition, TGF-β, and IL-13 levels, thereby resolving myocardial architectural disarray and preserving tissue cytoarchitecture (Jia et al., 2023). *Ex vivo*, ICA blocked TGF-β/Smad2 signaling to inhibit proliferation, EMT and stem-like properties in MDA-MB-231 breast cancer cells (Song et al., 2024). Beyond canonical profibrotic pathway modulation, ICA additionally ameliorated BLM-induced pulmonary fibrosis by downregulating cannabinoid receptor (CB) expression and dampening endocannabinoid signaling (Du et al., 2022).

#### 3.6.6 SIRT1 and cGAS/STING: neuroprotection (low evidence)

ICA confers neuroprotection primarily through SIRT1 activation and modulation of the cGAS/STING axis. It ameliorated amyloid pathologies (Jiang X. et al., 2019) and protected against oxidative neuronal injury (Zhang et al., 2010) through SIRT1 activation. In middle cerebral artery occlusion models, ICA induced peroxisome proliferator-activated receptor gamma coactivator 1-alpha (PGC-1α) expression in a SIRT1-dependent manner (Zhu et al., 2010). *Ex vivo*, it enhanced neuronal viability post-oxygen-glucose deprivation via MAPK/p38-mediated SIRT1 upregulation (Wang et al., 2009). Beyond canonical SIRT1 signaling, ICA downregulated cGAS, STING, and p-TBK1, thereby attenuating neuroinflammation and apoptosis in AD models (Lu et al., 2025), highlighting convergent protective pathways against neurodegeneration.

#### 3.6.7 Other signaling pathways (low evidence)

Wnt/β-catenin: ICA promoted osteoblast mineralization via Wnt/β-catenin activation (Wang Y. et al., 2018) and improved cognitive function in 3 × Tg-AD mice by enhancing glycolytic flux through this pathway (Liu J. et al., 2024). Conversely, it inhibited this cascade to stall cell cycle progression and induced apoptosis in SKOV-3 and MCF-7 cancer cell models (Fu et al., 2022).

PI3K/AKT: ICA attenuated MMP-3 and increased collagen II production in SW1353 chondrocytes by inhibiting PI3K/Akt/mTOR pathway (Chen et al., 2022). ICA induced mitochondrial apoptosis and G_2_/M phase arrest in MCF-7 cells by downregulating maternal embryonic leucine zipper kinase (MELK) and the PI3K/AKT signaling (Cheng et al., 2019). Similarly, ICA promoted apoptosis and reduced SKOV3 cell viability by inhibiting PI3K/Akt pathway (Wang et al., 2020). In rat models of perimenopausal depression, ICA rebalanced disrupted sex hormone levels by modulating neurotransmitter secretion and enhancing immune function, with these effects involving regulation of the PI3K/Akt pathway (Cao et al., 2019).

## 4 Safety profile and botanical–drug interaction update of ICA

### 4.1 Preclinical safety (low evidence)

According to TCM theories, processing Epimedii Folium with mutton oil is traditionally employed to reduce toxicity and potentiate effect. Recent zebrafish experiments verified that sheep-fat-processed botanicals yield a higher median lethal concentration and lower teratogenic rates than the raw botanicals (Fan et al., 2023). Although these findings imply that processing reshapes the toxicological profile, further studies are needed to clarify how processing affects ICA stability and metabolism and to validate safety in mammalian models. ICA is generally well tolerated in adult rodents and zebrafish under short-term exposure. Mice receiving 40 mg/kg ICA for 3 weeks showed no marked changes in body weight, white blood cell counts, or hepatic and renal function (Chen X. et al., 2023; Li et al., 2014). Adult zebrafish likewise developed no hepatic histopathological lesions after 15-day exposure (Zhong et al., 2019). By contrast, exposure to 10 µM ICA during early embryogenesis caused severe morphological malformations and decreased circulating T3 and T4 levels (Wu et al., 2023). Because thyroid hormone signaling is indispensable for embryonic development (Bagci et al., 2015; Yen, 2001), ICA likely induces developmental toxicity by disrupting thyroid hormone homeostasis. Notably, this teratogenic potential has not yet been validated in standard mammalian embryo-fetal development studies.

### 4.2 Clinical safety (high evidence)

Clinical data suggest that ICA has acceptable tolerability and a low incidence of adverse events in humans. A study enrolled 20 HCC patients who received ICT at 600–800 mg twice daily for 56 days. Hyperbilirubinemia was the most frequent event, and no grade ≥3 drug-related adverse reactions were recorded (Fan et al., 2019). A 24-month randomized, double-blind, placebo-controlled study in 100 late-postmenopausal women found that daily supplementation with 60 mg ICA plus 15 mg daidzein and 3 mg genistein did not alter serum estradiol levels or endometrial thickness (Zhang et al., 2007). These results indicate a low propensity for estrogen-like proliferative effects on reproductive tissues, unlike conventional estrogen receptor agonists. However, two of six participants withdrew on day 2 because of gastrointestinal intolerance at a daily dose of 1,680 mg ICA (Brown et al., 2019). Safety data remain sparse for vulnerable groups such as pregnant women, children, and older adults.

### 4.3 Botanical–drug interactions (low evidence)

Both ICA and its aglycone ICT influence key drug-metabolizing enzymes and efflux transporters, raising concerns over botanical-drug interactions. ICT acted as an inhibitor of CYP1A2, CYP2C9 and CYP3A4 (Liang and Zheng, 2014), while ICA induced CYP4A14 and elevated P-gp levels (Xu et al., 2014; Sun et al., 2015). Mechanistically, ICT irreversibly suppressed CYP2C9 through covalent binding (Chen X. et al., 2023). These regulatory effects could modify the pharmacokinetics of narrow therapeutic index drugs—including paclitaxel, tacrolimus, and cyclosporine A (Ando et al., 1998; Ring et al., 2005; Hauser et al., 1998). In addition, ICA co-exposure with botanicals such as bavachin has been associated with synergistic hepatotoxicity (Li Y. et al., 2024).

## 5 Current status of delivery systems

Between 2023 and 2025, multiple delivery platforms have been developed to improve ICA solubility, tissue-targeting capacity, and sustained-release performance across diverse disease contexts (see Table 8). Lipid micelles and PLGA microspheres were evaluated for chronic obstructive pulmonary disease, pulmonary fibrosis and lung cancer (Jiang and Somavarapu, 2025; Xiong et al., 2025). Cyclodextrin complexes and chitosan-modified nanoparticles were explored for hepatic applications (Ding et al., 2023; Fan et al., 2025). Bone-regenerative biomaterials—including hydrogels, magnesium-doped matrices, and hydroxyapatite scaffolds—have been tested for bone disorders and osteosarcoma (Li X. et al., 2024; Chen et al., 2024). Modified nanoliposomes and chitosan/PEG-PLGA composites showed therapeutic potential in neurological disorder models (Wang J. et al., 2025). Gelatin methacryloyl hydrogels and microneedle patches were investigated for reproductive diseases (Mao et al., 2025), while peptide-decorated nanoparticles were designed to mitigate ICA-induced myelosuppression (Xiao et al., 2024). These lipid, polymer and biomimetic carriers solved major problems in ICA delivery. Collectively, lipid-, polymer-, and biomimetic-based carriers address key physicochemical barriers of ICA delivery. Nevertheless, most formulations still exhibit suboptimal targeting precision, pronounced initial burst release, and limited translational readiness.

**TABLE 8:** The delivery systems for ICA (2023–2025).

Disease model	Carrier/technology
Chronic obstructive pulmonary disease	DSPE/PEG2000/DPPC micelle-in-microparticles with trehalose (Jiang and Somavarapu, 2025)
Pulmonary fibrosis	DSPE/PEG2000/DPPC micelles (Jiang and Somavarapu, 2024)
Metastatic lung cancer	PLGA microspheres (Xiong et al., 2025)
Lung cancer	TPGS/DPPC micelles (Jiang et al., 2025)
Osteosarcoma	Nano-hydroxyapatite/gelatin/polylactic acid fibrous membrane (Liu J. et al., 2023)
Osteosarcoma	Mg-doped mesoporous hydroxyapatite microsphere (Liu K. et al., 2024)
Liver diseases	HP- *γ*-cyclodextrin (Ding et al., 2023)
NASH	Chitosan and mannose modified PLGA/PEG nanoparticles (Fan et al., 2025)
Bone diseases	Nanoparticle/hydrogel hybrid system (Li X. et al., 2024)
Bone diseases	Magnesium doped- silicon based-nanoplatforms (Chen et al., 2024)
Bone diseases	Mussel-inspired hydrogel (Zeng et al., 2023)
Bone diseases	ICA/tannic acid nanodiamonds (Yu et al., 2023)
Bone diseases	Lactic-co-glycolic acid and silk fibroin microspheres (Zhang et al., 2023)
Bone diseases	p-phosphonatocalix [4]arene tetradodecyl ether (Luo et al., 2024)
Bone diseases	PLGA/PEG/PLGA hydrogels (Xu et al., 2025)
Bone diseases	Hydrogel (Zheng et al., 2025)
Bone diseases	Polydopamine modified covalent organic framework nanoparticles (Luo et al., 2025)
Bone diseases	Polycaprolactone scaffolds (Park et al., 2025)
Bone diseases	Titanium alloy scaffolds with chitosan hydrogel (Zhu et al., 2024)
Bone diseases	p (VCL-co-HEMA)-Based thermosensitive hydrogels (García-Sobrino et al., 2024)
Epilepsy	Nanoliposomes modified with CD47 mimicry peptide (Wang J. et al., 2025)
Vascular dementia	Chitosan hydrogel and PEG-PLGA nanoparticles (Li T. et al., 2024)
Reproductive diseases	Gelatin methacryloyl hydrogel (Mao et al., 2025)
Reproductive diseases	Silk fibroin (Fang et al., 2024)
Reproductive diseases	Chondroitin sulfate and polyvinylpyrrolidone K30 microneedle patches (Fang et al., 2025)
Myelosuppression	DSS6 peptide-modified nanoparticles (Xiao et al., 2024)

Abbreviations: DSPE, 1,2-distearoyl-sn-glycero-3-phosphoethanolamine; PEG, polyethylene glycol; DPPC, 1,2-dipalmitoyl-sn-glycero-3-phosphocholine; PLGA, Poly (lactic-co-glycolic acid); TPGS, D-α-tocopheryl polyethylene glycol 1000 succinate.

## 6 Discussion

### 6.1 Integrating traditional wisdom with modern pharmacological insights

Epimedii Folium was historically prescribed for osteoporosis, infertility, and general debilitation in TCM. Modern pharmacology corroborates these uses, showing that ICA exerts bone-protective, fertility-promoting, neuroprotective, and anti-fibrotic activities aligned with the its traditional profile. However, contemporary research overwhelmingly focuses on purified ICA as a single entity, largely overlooking how traditional processing alters its chemical landscape and how multicomponent synergy contributes to efficacy. Future investigations should therefore integrate classical processing principles and whole-botanical context, rather than treating ICA as an isolated small molecule.

### 6.2 The polypharmacology paradox: selectivity and therapeutic index

The polypharmacology of ICA presents both opportunity and translational liability. Unlike synthetic drugs engineered for single target, ICA interfaces with diverse receptors and signaling cascades. Its prenyl moiety enables hydrophobic docking to targets such as ERα and PDE5, while glycosyl groups permit hydrogen-bonding interactions that initiate pleiotropic regulation. This network-level activity plausibly explains the traditional utility of Epimedii Folium in complex, multisymptom syndromes. Yet, low molecular selectivity also raises concerns over off-target toxicity and narrow safety margins. Rather than being developed as a conventional single-indication drug, ICA is better conceptualized as a holistic multi-target modulator whose clinical niche lies in systemic rather than precision mono-therapies.

### 6.3 Translational gap and future directions

Despite robust preclinical activity in rodent models, ICA’s clinical translation remains hampered by critical bottlenecks. Most preclinical evidence relies on single-species, chemically induced, or ectopic xenograft models with limited human relevance, coupled with incomplete mechanistic validation—particularly a paucity of *in vivo* genetic rescue assays to confirm direct target engagement. Adding to this is a stark interspecies pharmacokinetic disparity: oral doses of 1–10 mg/kg achieved measurable systemic exposure in rodents (Xu Y. et al., 2017; Li Q. et al., 2022; Li et al., 2025), whereas a single 80 mg dose in humans yielded negligible plasma concentrations (Xu et al., 2007). Moreover, although zebrafish data indicates developmental toxicity, mammalian reproductive and chronic toxicity datasets remain sparse. ICA/ICT-mediated modulation of CYP450 enzymes and P-gp further introduces botanical–drug interaction risks, especially in patients on narrow therapeutic index medications. Collectively, gaps in model fidelity, mechanistic rigor, PK translatability, and safety underscore why existing clinical studies remain exploratory.

A registry review of ClinicalTrials.gov and ChiCTR (see Table 9) confirms this translational immaturity, revealing four recurring weaknesses. First, patient stratification is rudimentary; although pioneering HCC trials now incorporate composite biomarkers (AFP/TNF-α/IFN-γ; ChiCTR2200062675), such enrichment remains exceptional. Second, statistical power is low due to small cohorts, masking rare adverse events. Third, methodological quality is suboptimal—single-arm, non-randomized designs dominate, and even controlled trials often omit rigorous blinding. Fourth, dosing lacks standardization, with daily regimens fluctuating arbitrarily from 100 mg to 800 mg and formulations ranging from crude extracts to purified monomers.

**TABLE 9:** Global registered clinical trials of ICA or ICT.

NCT/ChiCTR no.	Phase	Indication	Intervention	Trial design
NCT02496949 (Fan et al., 2019)	I	Advanced HCC with Child-Pugh Class A or B	1,200 or 1,600 mg of ICT, dosing for 56 days	Single-arm
NCT02112123 (Brown et al., 2019)	I	Healthy participants	100–1,680 mg/day ICA for 5 days	Single-arm
NCT07076784 (Kim et al., 2026)	N/A	Inflammatory acne and atrophic acne scars	3% ICA solution twice daily for 2 months; Ctrl group: Topical application of 3% ethanol solution	Parallel
ChiCTR2300074565 (Shao et al., 2024)	Retrospective study	Unresectable HCC with CNLC Stage IIa-IIIb	ICT monotherapy or combined with PD-1/L1 and/or anti-VEGF therapy	Sequential
ChiCTR 2300077606	IV	Unresectable HCC	ICA + Sintilimab + Bevacizumab; Ctrl group: Sintilimab and bevacizumab	Parallel
NCT06900478	II	HCC at high risk of recurrence after resection	ICA + TACE	Single-arm
ChiCTR 2200059158	N/A	Neutropenia after chemotherapy for non-Hodgkin’s lymphoma	ICT + R-CHOP	Single-arm
ChiCTR 2200062675	III	Unresectable HCC with complicated disease condition, poor prognosis, and positive composite biomarkers at baseline	ICT + Cinobutanin tablets placebo Ctrl group: ICT soft capsule placebo + Cinobutalin tablets	Parallel
NCT05903456	II	Unresectable, non-metastatic HCC	ICT + TACE + Lenvatinib	Single-arm
ChiCTR 2300077907	N/A	Advanced pancreatic ductal adenocarcinoma	ICT + Gemcitabine + Nab-paclitaxel	Single-arm
ChiCTR 2300077987	N/A	Intermediate-advanced/critical resectable HCC	ICT + TACE + Lenvatinib	Single-arm
ChiCTR 2300077991	N/A	Intermediate and advanced HCC	ICT + TACE + TKI	Single-arm
ChiCTR 2400094250	IV	Had not received any systemic therapy for HCC group; Ctrl group: Had failed one or more lines of previous systemic therapy or who could not tolerate subsequent continuous therapy group	ICT	Non randomized control
ChiCTR 2500110058	II	Unresectable HCC	Interventional therapy + Sintilimab + Bevacizumab + ICT; Ctrl: Interventional therapy + Sintilimab + Bevacizumab	Non randomized control
ChiCTR 2500097382	N/A	Unresectable advanced biliary tract tumors	ICT + Adabelimab/Carilizumab + Cisplatin + Gemcitabine	Sequential
ChiCTR 2500100484	IV	Malignant biliary tract tumors	ICT + mFOLFOX chemotherapy	Single-arm
ChiCTR 2500101716	I	Health	ICT	Single-arm
ChiCTR 2600117381	N/A	Advanced HCC	Group 1: 6 capsules (600 mg) of *Epimedium* extract soft capsules once a day; Group 2: 9 capsules once a day; Group 3: 12 capsules once a day; Group 4: 6 capsules twice a day for 7 days	Non randomized control

Abbreviations: CNLC, china liver cancer staging; TACE, transcatheter arterial chemoembolization.

To bridge these limitations, a staged translational roadmap is warranted:

#### 6.3.1 Stage 1: strengthening preclinical foundations

Future preclinical work must mandate *in vivo* genetic rescue experiments and *in vitro* inhibitors/activators to confirm on-target effects. Formulation science should prioritize overcoming poor oral bioavailability and P-gp efflux. Furthermore, more research in vulnerable populations or those on narrow therapeutic index medications is also essential.

#### 6.3.2 Stage 2: standardizing clinical protocols and patient selection

Subsequent trials should adopt biomarker-guided enrichment, restricting enrollment to molecularly defined subgroups most likely to respond, and applying uniform dosing anchored in formal PK modeling of well-characterized ICA preparations.

#### 6.3.3 Stage 3: elevating clinical evidence quality

The field must transition from underpowered pilot reports to adequately powered, randomized, double-blind, placebo-controlled Phase III trials, shifting endpoints decisively toward hard clinical outcomes (e.g., fracture incidence in osteoporosis, overall survival in oncology) rather than surrogate markers alone.

## 7 Conclusion

As the principal bioactive flavonoid glycoside of *Epimedium* species, ICA bridges TCM theories and modern pharmacology. This review critically summarizes recent advances in ICA, including its structure–activity relationship, pharmacokinetics, pharmacological effects, safety profiles, clinical trials and delivery systems. Nevertheless, the translation of ICA from bench to bedside remains constrained by poor oral bioavailability and unresolved toxicological questions. Moving forward, research efforts must prioritize rationally designed combination regimens, alongside advanced biomaterial-based delivery systems to overcome ICA’s inherent biopharmaceutical limitations. Only through this integrated strategy can ICA’s polypharmacology be translated into robust, clinically meaningful outcomes.

## Glossary

ICA: Icariin
ED: Erectile dysfunction
TCM: Traditional Chinese medicine
ICA I: Icariside I
ICA II: Icariside II
ICT: Icaritin
ONFH: Osteonecrosis of the femoral head
OA: Osteoarthritis
GRK2: G protein-coupled receptor kinase 2
MELK: Maternal embryonic leucine zipper kinase
ALP: Alkaline phosphatase
OVX: Ovariectomy
GIOP: Glucocorticoid
AIA: Antigen-induced arthritis
***t***_**1/2**_: Terminal elimination half-life
HCC: Hepatocellular carcinoma
BMD: Bone mineral density
TRACP-5b: Tartrate-resistant acid phosphatase isoform 5b
CTX-I: C-telopeptide of type I collagen
DXM: Dexamethasone
BMECs: Bone microvascular endothelial cells
T1DM: Type 1 diabetes mellitus
BV/TV: bone volume
Tb.Th: trabecular thickness
Tb.N: trabecular number
Tb.Sp: trabecular separation
rBMSCs: Rat bone mesenchymal stem cells
ERα: Estrogen receptor α
BCNI: Bilateral cavernous nerve injury
ICP: Intracavernous pressure
MAP: Mean arterial pressure
CRC: Colorectal cancer
eNOS: Endothelial nitric oxide synthase
nNOS: Neuronal nitric oxide synthase
OVX: Ovariectomized
PGC-1α: Peroxisome proliferator-activated receptor gamma coactivator 1- alpha
CC: Corpus cavernosum
STZ: Streptozotocin
ECM: Extracellular matrix
DPPC: Dipalmitoylphosphatidylcholine
BLM: Bleomycin
EMT: Epithelial–mesenchymal transition
CB: Cannabinoid receptor
FN: Fibronectin
NASH: Non-alcoholic steatohepatitis
Col-1α: Collagen type I α
UUO: Unilateral ureteral obstruction
T2DM: Type 2 diabetes mellitus
VE-cad: Vascular endothelial cadherin
FSP-1: Fibroblast-specific protein 1
Nrf2: Nuclear factor erythroid 2-related factor 2
HO-1: Heme oxygenase-1
GLP-1R: Glucagon-like peptide-1 receptor
TPGS: D-α-tocopheryl polyethylene glycol succinate
AD: Alzheimer’s disease
PD: Parkinson’s disease
Aβ: Amyloid-β
A53T Tg: A53T α-synuclein transgenic
P-gp: P-glycoprotein
PDE5: Phosphodiesterase 5
TACE: Transcatheter Arterial Chemoembolization
STING: Stimulator of interferon genes
SHR: Spontaneously hypertensive rats
ROS: Reactive oxygen species
cGAS: Cyclic GMP-AMP synthase
AUC: Area under the plasma concentration–time curve
cGMP: Cyclic guanosine monophosphate
SNP: Sodium nitroprusside
