# Supplementary material for: Icariin: from preclinical promise to clinical reality—a critical evidence-based assessment
Source: Front Pharmacol. 2026 Sep 16;17:1832264. doi: 10.3389/fphar.2026.1832264 (PMC13624405; doi:10.3389/fphar.2026.1832264)
Supplement: Supplementary file 1 [file Table1.docx]

# Database-adapted Boolean search strategies for PubMed, Scopus and Google Scholar

Consistent composite Boolean combination formula across all databases: (#1 AND #2 AND #3) OR (#1 AND #2 AND #4) OR (#1 AND #3 AND #4) OR (#1 AND #2) OR (#1 AND #3) OR (#1 AND #4)

## 1. PubMed (MeSH + free text)

### Block #1 (Herb and active compounds)

(Epimedium[Title/Abstract] OR "Epimedii Folium"[Title/Abstract] OR icariin[Title/Abstract] OR "icariside II"[Title/Abstract] OR icaritin[Title/Abstract])

### Block #2 (Pharmacokinetics and chemical structure)

(structure[Title/Abstract] OR pharmacokinetics[MeSH Terms] OR bioavailability[Title/Abstract] OR absorption[MeSH Terms] OR distribution[MeSH Terms] OR metabolism[MeSH Terms] OR excretion[MeSH Terms])

### Block #3 (Diseases and pathological disorders)

(osteoporosis[MeSH Terms] OR "erectile dysfunction"[Title/Abstract] OR "reproductive disorders"[Title/Abstract] OR neurodegeneration[Title/Abstract] OR "brain injury"[MeSH Terms] OR "cerebral ischemia"[MeSH Terms] OR stroke[MeSH Terms] OR "cognitive impairment"[Title/Abstract] OR fibrosis[Title/Abstract] OR tumor[MeSH Terms] OR cancer[Title/Abstract])

### Block #4 (Safety, drug delivery and clinical research)

(safety[Title/Abstract] OR "Drug Delivery Systems"[MeSH Terms] OR "clinical study"[Title/Abstract])

## 2. Scopus (TITLE-ABS-KEY field restriction)

### Block #1

TITLE-ABS-KEY(Epimedium OR "Epimedii Folium" OR icariin OR "icariside II" OR icaritin)

### Block #2

TITLE-ABS-KEY(structure OR pharmacokinetics OR bioavailability OR absorption OR distribution OR metabolism OR excretion)

### Block #3

TITLE-ABS-KEY(osteoporosis OR "erectile dysfunction" OR "reproductive disorders" OR neurodegeneration OR "brain injury" OR "cerebral ischemia" OR stroke OR "cognitive impairment" OR fibrosis OR tumor OR cancer)

### Block #4

TITLE-ABS-KEY(safety OR "drug delivery" OR "clinical study")

## 3. Google Scholar (free text)

### Block #1

Epimedium OR "Epimedii Folium" OR icariin OR "icariside II" OR icaritin

### Block #2

structure OR pharmacokinetics OR bioavailability OR absorption OR distribution OR metabolism OR excretion

### Block #3

osteoporosis OR "erectile dysfunction" OR "reproductive disorders" OR neurodegeneration OR "brain injury" OR "cerebral ischemia" OR stroke OR "cognitive impairment" OR fibrosis OR tumor OR cancer

### Block #4

safety OR "drug delivery" OR "clinical study"
